# Variable partition of non-homogeneous ooplasm sets the stage for divergent potency of 2-cell stage blastomeres

**DOI:** 10.1093/molehr/gaag004

**Published:** 2026-02-04

**Authors:** Thomas Nolte, Reza Halabian, Steffen Israel, Yutaka Suzuki, Hannes C A Drexler, Wojciech Makalowski, Georg Fuellen, Michele Boiani

**Affiliations:** Max Planck Institute for Molecular Biomedicine, Münster, Germany; Faculty of Medicine, Institute of Bioinformatics, University of Münster, Münster, Germany; Max Planck Institute for Molecular Biomedicine, Münster, Germany; Department of Computational Biology and Medical Sciences, Graduate School of Frontier Sciences, University of Tokyo, Tokyo, Japan; Max Planck Institute for Molecular Biomedicine, Münster, Germany; Faculty of Biology, Institute of Experimental Biology, Adam Mickiewicz University, Poznań, Poland; Institute for Biostatistics and Informatics in Medicine and Ageing Research, Rostock University Medical Center, Rostock, Germany; Max Planck Institute for Molecular Biomedicine, Münster, Germany

**Keywords:** 2-cell embryo, blastocyst, embryo splitting, epiblast, ICSI, mass spectrometry, mouse, oocyte bisection, proteome, RNA-sequencing

## Abstract

Following fertilization in mice and humans, the first two blastomeres are not equivalent, but one produces more epiblast than the other (imbalance); therefore, they do not feature equal totipotency. Research into the causes has overlooked that the epiblast imbalance is preceded by a fertilization imbalance, since in nature, the spermatozoon fertilizes the oocyte preferentially in the animal hemisphere near the animal–vegetal midline (equator). We conceived a hypothesis that the two imbalances are linked to each other, and broke it down into testable predictions. If the two imbalances were interdependent, then changing the site of sperm entry into the oocyte should change the extent of the epiblast imbalance. Thus, we evened out the fertilization imbalance, using ICSI to fertilize mouse oocytes also in the vegetal hemisphere and the equator. Resultant embryos were split at the 2-cell stage, and the twin blastocysts originating from the sister blastomeres were analyzed. Against the similarity in mRNA levels of epiblast genes, twin blastocysts differed in epiblast function, as measured by NANOG protein expression and derivation of embryonic stem cells, and the epiblast imbalance was greater after oocyte fertilization at the equator. There is no simple way to explain the positional effect other than through differences in the molecular composition of the ooplasm, which, moreover, should also be apportioned variably at the first zygotic division. We tested these predictions by measuring the orientation of the first zygotic division regarding the ICSI site, and the composition of bisected oocytes’ hemispheres using half-cell proteomics. Since we found that the hemispheres have different compositions depending on the bisection axis, and the angle of the first division is variable, we propose that the variable partition of non-homogeneous ooplasm sets the stage for the epiblast imbalance. These results revive the role of the oocyte’s molecular architecture on embryogenesis in a mammalian species hitherto considered mostly regulative in development.

## Graphical Abstract

**Figure:**
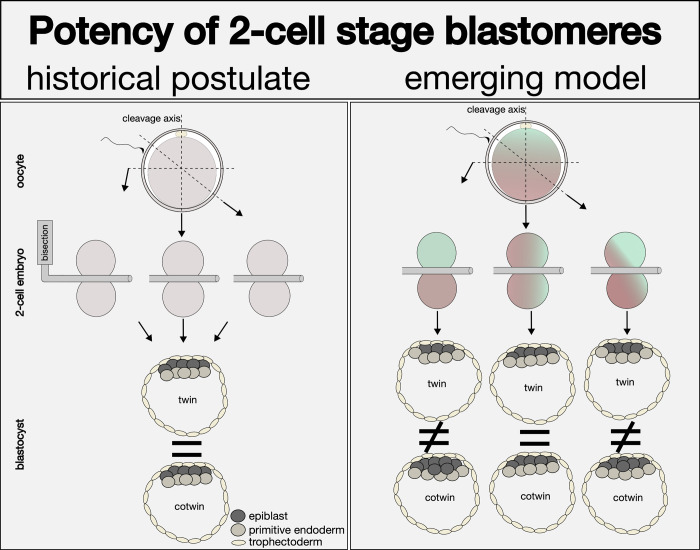
**It is proposed that if the ooplasm is not homogeneous and is apportioned variably at first zygotic division, the two blastomeres will be imbalanced in the production of epiblast at the blastocyst stage**.

## Introduction

Whether the first two blastomeres of the mammalian embryo are equal or not in terms of potency is a question that has fascinated biologists for decades. It was reported for the first time in 1959 that a single blastomere of a 2-cell embryo could develop into live newborn in mice, thereby supporting that cellular totipotency extended beyond the zygote stage (Tarkowski, 1959). In those days, it was not possible to separate the two blastomeres from each other and culture them *in vitro* as twins; thus, the approach was to destroy one of the two blastomeres inside the zona pellucida, and transfer the remaining blastomere to the female genital tract. If the orphan blastomere developed, it was assumed that the other would do the same. This was a generalization based on some blastomeres that succeeded, among others that did not at full development—a generalization also common to studies conducted in other species (reviewed in Boiani *et al.*, 2019). Later, as techniques improved in the 1980–1990s, it became possible to culture and examine both blastomeres in parallel, as twins (Tsunoda and McLaren, 1983; Togashi *et al.*, 1987; Wang *et al.*, 1997; Sotomaru *et al.*, 1998). When one of the twins succeeded at development while the other did not, it was assumed that the latter was an experimental error or a biological exception. The undemonstrated general assumption in the 2000s was that the potency of one blastomere was equal to that of the other blastomere.

However, one sometimes needs a second look and different approaches to realize that not all was as it first seemed. As it became possible to compare the sister blastomeres with each other on a large scale, it was noted that one blastomere produced more epiblast cells than the other (Casser *et al.*, 2017)—an ‘epiblast imbalance’. The epiblast is one of the three cell lineages of the embryo at the blastocyst stage, next to the trophectoderm and primitive endoderm. The number of epiblast cells matters, because it is the precursor of adult body tissues, and in mice, a minimum of four epiblast cells (NANOG protein-positive) is required for a blastocyst to be viable (Morris *et al.*, 2012). In extreme cases, the epiblast originated from only one of the two sister blastomeres, while the amounts of trophectoderm and primitive endoderm were balanced between sister blastomeres (Casser *et al.*, 2017). Additional independent studies in mouse and human led to similar observations (Krawczyk *et al.*, 2021; Maemura *et al.*, 2021; Junyent *et al.*, 2024). In other words, each 2-cell blastomere was totipotent enough to form a blastocyst with all three lineages, but epiblast cells were not always enough—in number—to support full development. The root cause of the epiblast imbalance could lie in regulative processes unfolding differently in the cell progenies of the two blastomeres, or upstream factors possibly related to the differential allocation of maternal factors into the blastomeres. Irrespective of which possibility turns out to be true, a precondition to investigate these possibilities is to rely on unbiased biological material, and therein lies a catch: naturally fertilized (NF) oocytes are biased as there is an imbalance of fertilization. Spermatozoa tend to penetrate the mouse zona pellucida more frequently in the animal (A) hemisphere of metaphase II (MII) oocytes, as defined by the location of the maternal chromosomes (spindle), owing to the greater space created by the first polar body between oolemma and zona. However, in that region where the first polar body is extruded, the oolemma is less amenable to sperm fusion. The net result is that the oocyte is fertilized more frequently in the A hemisphere and at the equator than in the opposite (vegetal, V) hemisphere (Motosugi *et al.*, 2006; Mori *et al.*, 2021).

We hypothesized here that the epiblast imbalance of mouse monozygotic twins is linked to the initial fertilization imbalance. In order to test this hypothesis, we broke it down into three testable predictions. First, if the two imbalances were interdependent, then changing the site of sperm entry in the oocyte should change the extent of the epiblast imbalance. Second, if epiblast imbalance varied with the site of fertilization, then there would be no simple way to explain it, except by invoking differences in the molecular composition of the ooplasm. Third, if these ooplasmic differences existed, then they should also be apportioned variably at the first zygotic division. To test these predictions, not necessarily in the order listed above, we applied ICSI (Kimura and Yanagimachi, 1995) to even out the natural fertilization bias in the A hemisphere and, instead, also fertilized MII oocytes in the V hemisphere and in the cortex around the A–V midline (equator). After the ICSI, we quantified epiblast production by the sister blastomeres using multiple assays, observing that imbalance indeed varied with the site of fertilization. Since fertilization sites also influenced the orientation of the first zygotic division even before epiblast production, we simulated division by mechanically bisecting the oocyte. Thus, we analyzed the hemispheres of MII oocytes that were bisected manually along the A–V axis or the equator, using half-cell proteomics. The results show that hemispheres have different protein compositions depending on the bisection axis, and since the angle of the first division is variable, this provides a link between the fertilization imbalance and the epiblast imbalance. We are not claiming that this is the sole key to explaining divergence in potency between the two blastomeres, nor that initial positional information retains its original strength throughout preimplantation. More likely, divergence in potency is governed by a complex interplay of processes rather than a single event or mechanism, and initial positional information fades as development progresses. However, after the results of this study, it is no longer possible to maintain that polarity of mouse oocytes (and, by extension, mammalian oocytes) is functionally irrelevant, and that no traces of positional information persist at the blastocyst stage. We have drawn our conclusions from a mouse study, but since ICSI is a widespread practice in assisted human reproduction, we also discuss if and how our findings may be pertinent to human ICSI.

## Materials and methods

### Ethics statement and animal housing

Mice were used for experiments according to the ethical approval issued by the Landesamt für Natur, Umwelt und Verbraucherschutz of the state of North Rhine-Westphalia, Germany (Permit number 81-02.04.2020.A405, ‘Die Zuteilung der zygotischen Totipotenz in den ersten Blastomeren: Eine Untersuchung der Ursprünge und Mechanismen im Mausmodell’). Except for the retrieval of oocytes, the experiments were conducted *in vitro*. The experimental unit was the oocyte. All mice used were maintained in individually ventilated cages in the animal facility of the MPI Münster, with a controlled temperature of 22 °C, a 14/10 h light/dark photoperiod and free access to water and food (Teklad 2020SX, ENVIGO RMS GmbH, Düsseldorf, Germany). Procedures used in this study followed the ethical guidelines of the FELASA (Federation of the European Laboratory Animal Science Associations) and the ARRIVE (Animal Research: Reporting of *In Vivo* Experiments) reporting guidelines.

### Collection of oocytes

Eight-week-old B6C3F1 females, reared in house, were primed with 10 I.U. each of pregnant mare serum gonadotropin—PMSG (Pregmagon, Ceva Tiergesundheit, Düsseldorf, Germany) and hCG (Ovogest, MSD Tiergesundheit, Unterschleißheim, Germany) injected intraperitoneally 48 h apart at 5 pm, and then euthanized by cervical dislocation to collect MII oocytes from oviducts. Cumulus cells were removed in hyaluronidase (CAT No. 151271, ICN Biomedicals, Costa Mesa, CA, USA) 50 I.U./ml in CZB medium buffered with HEPES (HCZB; Cavaleri *et al.*, 2006). HCZB contained 0.2% w/v polyvinylpyrrolidone (PVP, 40 kDa; CAT No. 529504, Calbiochem, EMD Biosciences, La Jolla, CA, USA) in place of bovine serum albumin (BSA). After removing the cumulus cells, MII oocytes were kept in 500 µl of α-MEM medium (CAT No. M4526, Sigma-Aldrich Chemie GmbH, Taufkirchen, Germany) containing 0.2% w/v BSA (Probumin, Milllipore, Kankakee, IL, USA) and 50 µg/ml gentamicin, in a Nunc 4-well plate (CAT No. 176740, Thermo Scientific, Bremen, Germany) without oil overlay at 37 °C under 6% CO_2_ in air, until further processing.

### Collection of sperm and cryopreservation

Cauda epididymides of two CD1 or OG2 (Tg(Pou5f1-EGFP)2Mnn, JAX 004654) male mice aged 3 months were collected, and poked with a 27-gauge needle. Spermatozoa were allowed to swim up in ∼ 1 ml Whittingham medium (Fraser and Drury, 1975) supplemented with 3% w/v BSA (CAT No. A3311, Sigma-Aldrich Chemie GmbH). Swim up took place across a distance of ∼ 1.5 cm for 30 min under a humidified atmosphere of 6% CO_2_ in air at 37 °C. The upper layer (∼ 200 μl) of swim-up medium was collected and incubated under pre-equilibrated mineral oil (CAT No. M8410, Sigma-Aldrich Chemie GmbH), to allow for sperm capacitation. After 1 h, the 200 μl drop of medium containing the spermatozoa was centrifuged (200 ×*g*) and then resuspended in half the volume of Whittingham medium. Aliquots of 5 µl of sperm suspension were placed at −80°C for 1 week before storing in liquid nitrogen, until use.

### ICSI and embryo culture

The ICSI was conducted on a glass-bottomed dish on the stage of a Nikon TE2000-U microscope fitted with Nomarski optics, a Narishige micromanipulator (Model NT-88NE), and a piezo drill (PrimeTech Ltd., Ibaraki, Japan). The room temperature was 27 °C. The sperm heads were microinjected into the oocytes using a blunt-end glass needle (inner diameter 6–7 µm, outer diameter 8–9 µm) filled with 2–3 μl mercury at the tip. Injection volumes were controlled using a Gilmont GS-1200 micrometer syringe operated manually. At 14–15 h post-hCG, the MII oocytes were held in HCZB medium during the ICSI, following our micromanipulation protocol, in which the holding pipette is on the right-hand side and the ICSI pipette comes from the left, except that this time, the oocytes were also rotated to consistently inject the sperm head at 3 o’clock with the MII spindle at 3 o’clock (ipsilateral ICSI), 9 o’clock (contralateral ICSI), or 12 o’clock (equatorial ICSI). After a 10-min recovery time from the microinjection, fertilized oocytes were transferred to 500 µl of potassium (K) simplex optimization medium containing amino acids (KSOM(aa)) in a Nunc 4-well plate (CAT No. 176740, Thermo Scientific), without oil overlay at 37 °C under 6% CO_2_ in air. KSOM(aa) was manufactured in house following the original recipe (Summers *et al.*, 2000) and contained also 0.2% (w/v) BSA (Probumin, Milllipore) and gentamicin (50 µg/ml). The above ICSI procedure was modified when the oocytes had to be kept immobilized on the stage of the micromanipulator to determine the relationship between site of ICSI and angle of first zygotic cleavage (Nolte *et al.*, 2025). Instead of multiple oocytes, only one oocyte was injected per round of ICSI. Instead of the glass-bottomed dish a plastic dish was used, the HCZB medium was added with 25% (v/v) of KSOM(aa) medium, and the micromanipulation drop was overlaid with oil to preserve osmolarity. The injected oocyte was kept in the ICSI dish under gentle constant suction from the holding pipette, for 24 h, at a temperature of 37 °C maintained via a Thermo Plate (TOKAI HIT Co. Ltd., Shizuoka-ken, Japan). At the end of the 24-h period, pictures were taken.

### Splitting of 2-cell embryos and culture of individual blastomeres to blastocyst

Two-cell embryos arising in the time frame from 20- to 22-h post-ICSI were removed from the culture to perform splitting from 22- to 24-h post-ICSI. These embryos were transferred in groups of 12 to a micromanipulation drop on the stage of a Nikon Eclipse TE2000-U inverted microscope fitted with Nomarski optics, and holding and splitting needles in place. The splitting medium consisted of 0.2 mM D(+) glucose, 0.2 mM pyruvate, 10 mM lactate, 0.5% w/v BSA (CAT No. A3311, Sigma-Aldrich Chemie GmbH), in 0.9% w/v sodium chloride, as described previously (Casser *et al.*, 2017). We also made the splitting medium slightly hypertonic by using 95% of the water volume. The splitting tool was a TransferTip (ES) needle operated by a CellTram Vario (Eppendorf SE, Hamburg, Germany). The 2-cell embryo was rotated using the holding and splitting needle to align the cleavage plane with the common axis of the two pipettes. The 2-cell embryo was firmly held in place with the holding pipette by applying negative pressure (suction). The TransferTip was used to initially make a slit in the ZP at one pole and then press the ZP gently at the equatorial region, causing one blastomere to be squeezed out. Suction in the holding pipette was then reduced and the other blastomere came out by performing the same procedure, except that the needle was pressed against the ZP below the equatorial region. When all 12 embryos had been split, which normally took 8–10 min, the pairs of blastomeres were collected and transferred to another medium/vessel using a bent (≈ 60° angle), mouth-operated pipette with a flame-polished tip. The individual blastomeres allocated individually to culture in 75 µl KSOM(aa) medium in a 96-well plate with round bottoms (CAT No. 163320, Thermo Scientific), without oil overlay, at 37 °C under 6% CO_2_ in air. Culture under these conditions took place for an additional 72 h until the blastocyst stage.

### Transcriptome analysis of blastocysts

Single blastocysts were lysed in 10X Lysis Buffer (CAT No. 635013, TaKaRa Bio Inc., Kusatsu, Shiga, Japan), deposited in one 384-well plate (Mosquito^®^), for a total of 78 blastocysts (36 intact, 42 twins from 21 pairs). The intact blastocysts were defined as those in which the 2-cell embryo was not split (unlike the twins). The RNA was then extracted and converted to cDNA using a SMART-Seq Single Cell Kit (CAT No. 634470, TaKaRa Bio Inc.). Sequencing libraries were prepared using the Illumina Nextera XT DNA Library Preparation Kit (CAT No. FC-131-1024, Illumina Japan, Minato-ku, Tokyo, Japan). Libraries were sequenced on an Illumina NovaSeq 6000 platform to obtain a dataset of 2.9 ± 1.1 million total mapped reads per library (100-base single-end reads).

Of the initial 78 samples, 8 were excluded from the analysis based on quality reports from the sequencing lab. Consequently, transcriptomic analysis was performed on the remaining 70 samples. To quantify gene expression level, Salmon (v1.10.3) (Patro *et al.*, 2017) was used. The following reference files were downloaded from GENCODE (https://ftp.ebi.ac.uk/pub/databases/gencode/Gencode_mouse/release_M36/) on 24 January 2025: gencode.vM36.pc_transcripts.fa.gz, gencode.vM36.lncRNA_transcripts.fa.gz, gencode.vM36.primary_assembly.annotation.gtf.gz, GRCm39.primary_assembly.genome.fa.gz.

To build the index, protein-coding and lncRNA transcript FASTA files were combined into a single reference file, and decoy sequences were generated using the corresponding genome FASTA. Indexing was performed using salmon index with default settings and the additional options -d decoys.txt-gencode.

Transcript quantification was subsequently carried out using salmon quant with the following parameters: —libType A—unmatedReads—useVBOpt—seqBias—gcBias—writeQualities—geneMap ‘GTF’ —writeUnmappedNames. The output from Salmon represented expression levels in transcripts per million (TPM).

Following quantification, an additional six samples were excluded due to low mapping rates (<35%) identified during quality control. To reduce noise in downstream analyses, only genes with TPM >1 in at least three samples were retained.

Transcript-level quantifications were then aggregated to the gene level using the R package ‘tximport’ (Soneson *et al.*, 2015). ENSEMBL gene IDs were mapped to gene names using the org. Mm.eg.db annotation package (Carlson,  2018). To facilitate downstream processing, genes were sorted based on their geometric mean TPM values across all samples, from highest to lowest. Therefore, the resulting gene-level count matrix, comprising 19 341 genes across 64 blastocyst samples (26 intact and 38 twin embryos), was used for subsequent analysis. The above filtering steps are summarized in Supplementary Table S1.

### Analysis of cell lineage allocation of blastocysts

Seventy-two hours after 2-cell embryo splitting, the twin blastocysts were analyzed by performing either a DNA staining with Hoechst 33343 (1 µg/ml) to count all nuclei, or an immunostaining to distinguish the nuclei of trophectoderm, epiblast, and primitive endoderm cells, as described in our previous work (Schwarzer *et al.*, 2012; Casser *et al.*, 2017). Embryos were fixed in 1.5% paraformaldehyde in PBS/Triton X-100 0.1%/PVP 0.1% for 30 min at ambient temperature. After washing in PBT (PBS/Tween-20 0.1%/PVP 0.1%) for 20 min, unspecific binding sites were blocked by incubating in blocking buffer (PBS/donkey serum 5%/BSA 2%/Glycine 2%/Tween-20 0.1%) for at least 2 h at 4 °C. The following primary antibodies were applied simultaneously to the samples overnight at 4 °C for analysis of cell lineage allocation: anti-CDX2 mouse IgG1 (CAT. no. CDX2-88Emergo Europe, The Hague, The Netherlands), anti-NANOG rabbit IgG (CAT. no. REC-RCAB0002P-F, Cosmo Bio, Tokyo, Japan), and anti-SOX17 goat IgG (CAT No. AF1924, R&D Systems, Minneapolis, MN, USA) in dilutions of 1:200, 1:2000, and 1:100, respectively. Appropriate Alexa Fluor-tagged secondary antibodies (Invitrogen) were matched to the primaries and incubated for 2 h at room temperature. For microscopy, twin pairs were placed in 5 µl drops of PBS on a 50-mm thin-bottom plastic dish (Greiner Bio-One, Lumox hydrophilic dish; Frickenhausen, Germany) and overlaid with mineral oil (CAT No. M8410, Sigma-Aldrich Chemie GmbH). Images were captured on the stage of an inverted microscope (Eclipse 2000-U; Nikon, Düsseldorf, Germany) fitted with a spinning disk confocal unit (Ultra View RS3; Perkin-Elmer LAS, Jügesheim, Germany). A Nikon Plan Fluor 20/0.75 N.A. multi-immersion objective was used. Twenty optical sections per embryo were captured using a Hamamatsu ORCA ER digital camera (Hamamatsu Photonics KK, Shizuoka, Japan). Maximum projections were analyzed with Fiji (Schindelin *et al*., 2012).

### Derivation of ES cells from blastocysts

After fertilization with OG2 spermatozoa, which conferred the blastocysts with GFP-fluorescence in the epiblast compartment, we transferred twin blastocysts (72 h after 2-cell embryo splitting) individually onto a feeder layer of γ-ray-inactivated (30 *Gray*) mouse embryonic fibroblasts (C3H background) grown to confluence in 96-well plates (flat bottom) previously, using our adaptation (Boiani *et al.*, 2002) of the outgrowth method (Armant *et al.*, 1986). The medium consisted of high-glucose Dulbecco’s modified eagle medium with high glucose (CAT No. D5671, Sigma-Aldrich Chemie GmbH) supplemented with 15% heat-inactivated fetal bovine serum (BioWest, Nuaillé, France), GlutaMAX 1X (CAT. No. 35050-038, Gibco at Thermo Scientific), 1x penicillin/streptomycin (CAT. No. P4333, Sigma-Aldrich Chemie GmbH), 1× nonessential amino acids (CAT. No. M7145, Sigma-Aldrich Chemie GmbH), 0.1 mM mercaptoethanol (CAT No. 31350-010, Gibco at Thermo Scientific), 1000 units ml^−1^ LIF (produced in-house), and inhibitors of ERK1/2 and glycogen synthase kinase 3 (CHIR99021 1.5 μM, PD0325901 0.5 μM). Blastocysts that attached to the feeders formed trophoblastic outgrowths on day 3–4 after transfer to feeders. The outgrowths were trypsinized with 0.25% trypsin–EDTA (Gibco, Gaithersburg, MD, USA) and reseeded onto fresh feeder cells on a 96-well plate. Given the use of OG2 spermatozoa for ICSI, the ES cell colonies were GFP fluorescent. Colonies in each well were counted 5–6 days after initial plating of the dissociated outgrowths, using ‘Operetta’ high-content imager system (PerkinElmer). Images were acquired in GFP channel (488 nm) and processed in Harmony 4.1 software and/or Columbus version 2.6.0.

### Oocyte bisection and half-cell proteomics

MII oocytes were incubated in Hoechst 33342 (1 µg/ml) dissolved in α-MEM medium containing 0.2% (w/v) BSA and 50 µg/ml gentamicin, for 15 min at 37 °C under 6% CO_2_. This way, the spindle could be later visualized under UV light, allowing for consistent orientation of the oocytes relative to the spindle position. In some cases, MII oocytes were co-stained with MitoTracker Green (CAT No. M7514, Thermo Scientific) at a final concentration of 0.1 μM in α-MEM. Immediately prior to bisection, oocytes were deprived of the zona pellucida by treatment with acidic Tyrode solution. Bisections took place in a drop of micromanipulation medium on a glass-bottomed dish on the stage of the micromanipulator (same as used for ICSI). The micromanipulation medium consisted of a mixture of 10% α-MEM and 90% HCZB (v/v), supplemented as described, added with 5 µM Latrunculin B (CAT No. AG-CN2-0031-M001, AdipoGen Life Sciences, distributed by BIOMOL, Hamburg, Germany). The room temperature was 27 °C to facilitate the depolymerization of the actin cytoskeleton by Latrunculin B. Each MII oocyte was oriented so as to have the spindle at the 12 o’clock position in case of equatorial bisection conducted to separate A and V hemispheres, or 3 o’clock position in case of meridional bisection conducted to separate ‘left’ and ‘right’ hemispheres. Bisection was operated using a borosilicate capillary with O.D. 15 µm at the tip, connected to a piezo device (same as used for ICSI), but the capillary was not filled with mercury. The capillary was pressed downward to divide the oocyte into two parts. Usually, the two oocyte-halves remain connected by a thin cytoplasmic bridge, which was severed with the help of piezo pulses (Video 1, Video 2). Oocytes were processed in batches of 10 at a time in ∼20 min, keeping the two halves of the same oocyte next to each other but far enough from those of other oocytes. The two halves were collected keeping track of the source oocyte and of which half was A, V, left or right relative to the spindle.

The oocyte halves were processed for proteomics largely following the method of Zhang *et al.* (2025) with some modifications. Briefly, halves were collected in 8 μl lysis and digestion buffer deposited in autosampler HPLC vials (Cat. No. 176004434, QuanRecovery, Waters, Eschborn, Germany). The composition of the buffer was: 0.2% *n*-Dodecyl-β-d-Maltoside, 100 mM HEPES, and 20 ng/μl sequencing grade modified Trypsin (Promega). Digestion was conducted for 2 h at 37 °C. Nano liquid chromatography–tandem mass spectrometry (nanoLC–MS/MS) analysis was performed on a timsTOF pro mass spectrometer (Bruker Daltonics, Bremen, Germany) coupled to a nanoElute 2 ultra-high-performance liquid chromatography (UHPLC) system via a CaptiveSpray nano-electrospray ion source. Peptide samples were separated using a fused silica capillary column with integrated emitter tip packed with 1.9 μm/120 Å C18 resin (25 cm L×360 µm OD×75 µm ID; Protum*Link* core, Jena, Germany). Chromatography was accomplished using a 60-min gradient (total run time) from 4% to 20% buffer B (0.1% formic acid in acetonitrile) over 42 min, from 20% to 37% buffer B over 6 min and from 37% to 85% buffer B in 4 min, followed by a 8-min wash-out at 85% buffer B (buffer A: 0.1% formic acid in water; flow rate: 300nl/min; column temperature: 50 °C). Mass spectrometry data were recorded in diaPASEF mode, scanning precursors across an m/z range of 100–1700. Other settings were slightly adapted as well (fragmentation mass range with m/z 400.0–1193.0; ion mobility range 0.6–1.6 Vs/cm2; ramp time: 166.0 ms, accumulation time: 100 ms). Raw data were analyzed using Spectronaut 20 (Biognosys) in directDIA mode (directDIA+ Deep). Data were searched against the mouse UniProt reference database (version from January 2022), Carbamidomethylation on cysteine residues was set as fixed modification, while methionine oxidation and protein N-terminal acetylation were defined as variable modifications. Enzyme specificity was set to trypsin, allowing for a maximum of two missed cleavages. A minimum peptide length of 7 amino acids was required. Precursor filtering was set to perform based on Q-values; protein LFQ method was set to automatic. The mass spectrometry proteomics data have been deposited in the ProteomeXchange Consortium via the PRIDE repository (Perez-Riverol *et al.*, 2025) (http://proteomecentral.proteomexchange.org), with the dataset identifier PXD067319.

### Study design, statistical methods, and data analysis

The main hypothesis of this study was broken down into three testable predictions. If the fertilization imbalance and the epiblast imbalance were interdependent, then changing the site of sperm entry in the oocyte should change the extent of epiblast imbalance (prediction 1). If this positional effect were true, then the molecular composition of the ooplasm should be non-homogeneous (prediction 2), and these internal differences should be apportioned variably at first zygotic division (prediction 3). The embryos from the three ICSI sites were mainly compared with each other, occasionally with embryos fertilized *in vivo*. The sample sizes were not calculated *a priori*. No blinding was applied and both the experimenters and data analysts were aware of the group allocation throughout the entire experiment. Nuclear fluorescent signals after Hoechst 33342 staining were evaluated from entire stacks of confocal sections for each blastocyst using ImageJ software. Assuming that one nucleus corresponds to one cell, the total cell counts of twin and intact blastocysts were presented as box plots with superimposed dots indicating individual data points. Nuclear fluorescent signals of twin blastocysts after triple immunostaining (CDX2, SOX17, NANOG) were evaluated in the same way as those of Hoechst 33342. The number of ES cell colonies derived from twin and cotwin blastocysts was determined using Operetta high-content imager system. Whether cell nuclei or ES cell colonies, the paired counts of twin and cotwin were presented as *X*–*Y* scatter plots, consistently assigning the member of each pair with the lower count (twin ‘a’) to the *X*-axis and the member with the higher count (cotwin ‘b’) to the *Y* axis. In the case of mRNA values (TPM) obtained from the transcriptome analysis, assignment to ‘a’ or ‘b’ was made on the basis of the expression levels of *Actb* (Giebelhaus *et al.*, 1983, 1985). Statistical analysis of the twin–cotwin comparisons was made using non-parametric tests (Wilcoxon test, Spearman linear correlation) performed with the JMP Pro software v.18 (SAS Institute GmbH, Heidelberg, Germany). The significance level was set at *P* < 0.05. Gene ontology (GO) analysis of the transcriptomes was performed either considering or disregarding the a–b ranking. Considering the a–b ranking, TPM levels were expressed as scatter plots as described, except that the TPM values were first converted to base-2 logarithm to handle the much wider expression range of the TPMs (0–10^4^) compared to the cell counts (0–10^2^). Disregarding the a–b ranking, absolute differences of gene expression (TPM values) between blastocysts were ranked and fed into REVIGO (Supek *et al.*, 2011) for GO analysis. In the case of the proteome analysis of bisected oocytes, distribution analysis and principal component analysis (PCA) were performed using JMP. To evaluate the proteins that are differently abundant in the A versus V or left versus right halves of the oocytes, and thereby infer the proteomic non-homogeneity within individual MII oocytes, protein-level variability was quantified as follows. Total variance (SS) was partitioned into between-oocytes (SSbo) and within-oocytes (SSwo). SSwo/SS ratios were calculated using Excel. A threshold of 0.80 was determined empirically based on the observation that SSwo/SS ≥0.80 allowed to resolve the A and V halves in PCA. Proteins with the highest SSwo/SS ratios (≥0.80) were subjected to overrepresentation analysis using *Enrichr* (Chen *et al.*, 2013; Kuleshov *et al.*, 2016; Xie *et al.*, 2021).

## Results

### An ICSI model that allows one to test the link between epiblast imbalance and fertilization imbalance

**Figure 1.**
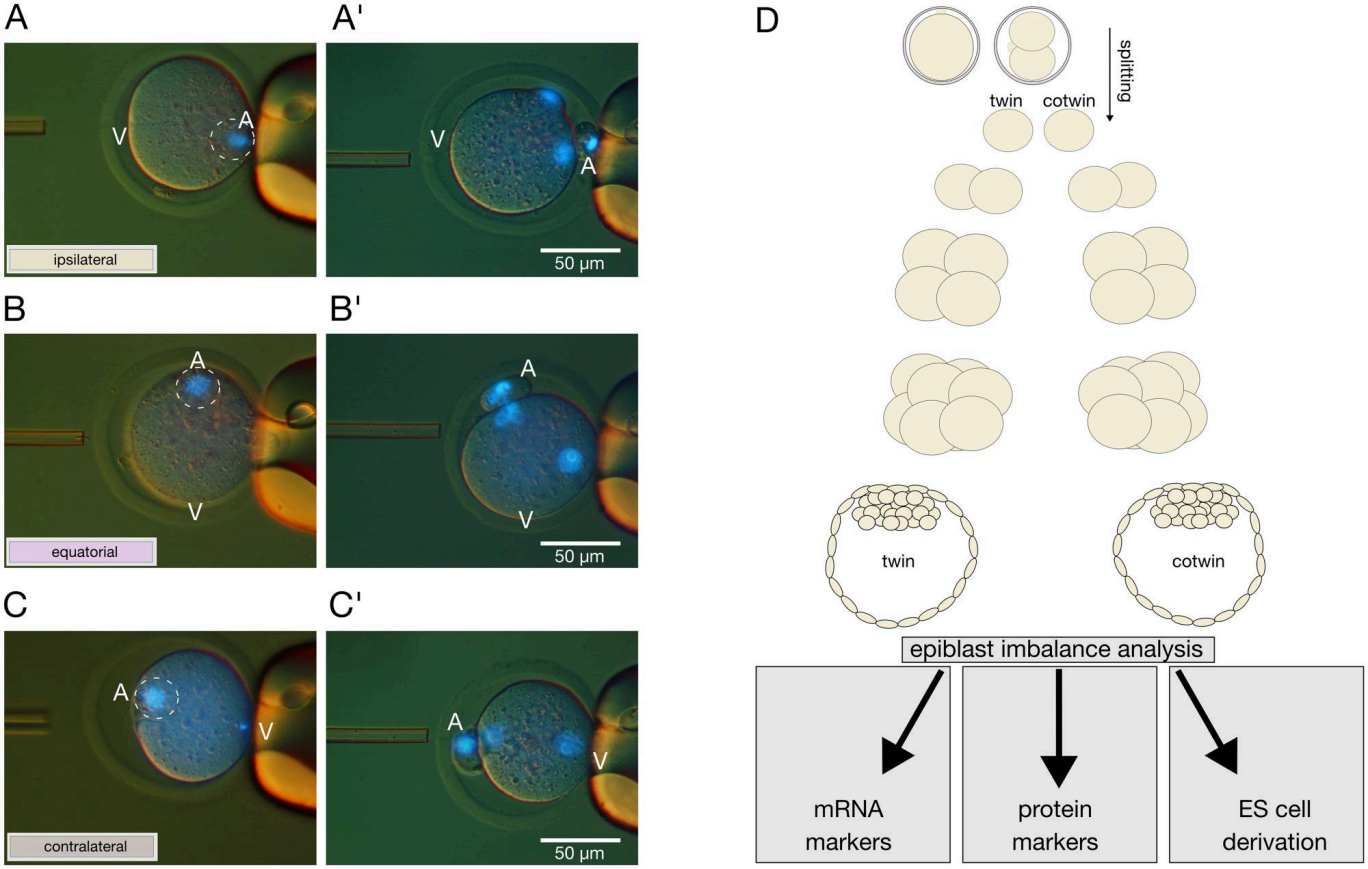
**Combined application of ICSI and embryo splitting to test the link between the epiblast imbalance in embryos and the fertilization imbalance in oocytes.** (**A, B, C**) Metaphase II (MII) mouse oocytes were held in position with a holding pipette on the right-hand side, and injected with a single sperm head in the region ipsilateral (**A**) or contralateral (**C**) to the spindle (encircled) or half-way in between (equatorial, **B**). **A’, B’, C’**: a second polar body had been extruded and pronuclei had begun to form 2–3 h after ICSI. Note: ‘ipsilateral’ = animal (A) pole, ‘contralateral’ = vegetal (V) pole. Blue color in photographs: DNA staining with Hoechst 33342 (1 µg/ml). (**D**) The embryos cultured *in vitro* were split at the 2-cell stage and the two halves were cultured further to blastocyst, keeping track of the original pair associations. Twin blastocyst pairs were allocated to three assays: transcriptome analysis (RNA sequencing), identification and quantification of cell lineages by protein markers (immunofluorescence), and functional evaluation of epiblast imbalance by means of embryonic stem (ES) cell derivation.

The premise that the sister 2-cell blastomeres produce different amounts of epiblast because the oocyte was fertilized preferentially in the animal (A) hemisphere (see Introduction) predicts that the epiblast imbalance should be evened out if half of the oocytes were fertilized in the other (vegetal, V) hemisphere. This alternative being naturally improbable (Motosugi *et al.*, 2006; Mori *et al.*, 2021), we imposed it experimentally by ICSI, and for additional resolution, we also fertilized the oocytes in the cortex around the A–V midline (equator), thereby creating three groups (Fig. 1A–C). Since the ICSI and holding micropipettes are fixed to the micromanipulator, it was the oocytes that were rotated, so as to have the meiotic spindle sitting at a 3, 9, or 12 o’clock position when introducing a single sperm head always from the 9 o’clock position toward the 3 o’clock position. In doing so, the sperm head was deposited right where the spindle is (ipsilateral), on the opposite side (contralateral), and halfway (equator). In terms of hemispheres, the oocytes were fertilized in the A hemisphere after ipsilateral ICSI, and in the V hemisphere after contralateral ICSI (Fig. 1A–C). Each round of ICSI included all three groups in approximately equal proportions (⅓), thereby minimizing the batch effects. We used the same batch of spermatozoa throughout the study for additional consistency; it was from the CD1 strain, unless otherwise stated, and aliquoted and cryopreserved for the purpose.

Two hundred MII oocytes (B6C3F1) were injected for each region with uniform survival rates (84 ± 8%). The oocytes invariably extruded a second polar body and formed two pronuclei irrespective of the ICSI site (Fig. 1A’–C’). We did not notice anything unusual about the pronuclear ICSI oocytes (zygotes), except perhaps that the fertilization cones were not as prominent as those of NF zygotes retrieved from oviducts after mating (NF oocytes). The zygotes were cultured to the 2-cell stage in three separate pools (ipsilateral, equatorial, and contralateral ICSI). From the 2-cell stage onward, the embryos were cultured individually in 96-well plates, either as intact embryos or after splitting had been conducted as per our established procedure (Casser *et al.*, 2017) (Fig. 1D), keeping track of the original pair associations. Members of the same pair after splitting are referred to as twin and cotwin. The blastocyst rate of intact embryos at 96 h post-ICSI was 42.6 ± 22.1% and 40.0 ± 19.7% after contralateral and equatorial ICSI, respectively, and only marginally lower after ipsilateral ICSI (38.2 ± 16.0%).

**Figure 2.**
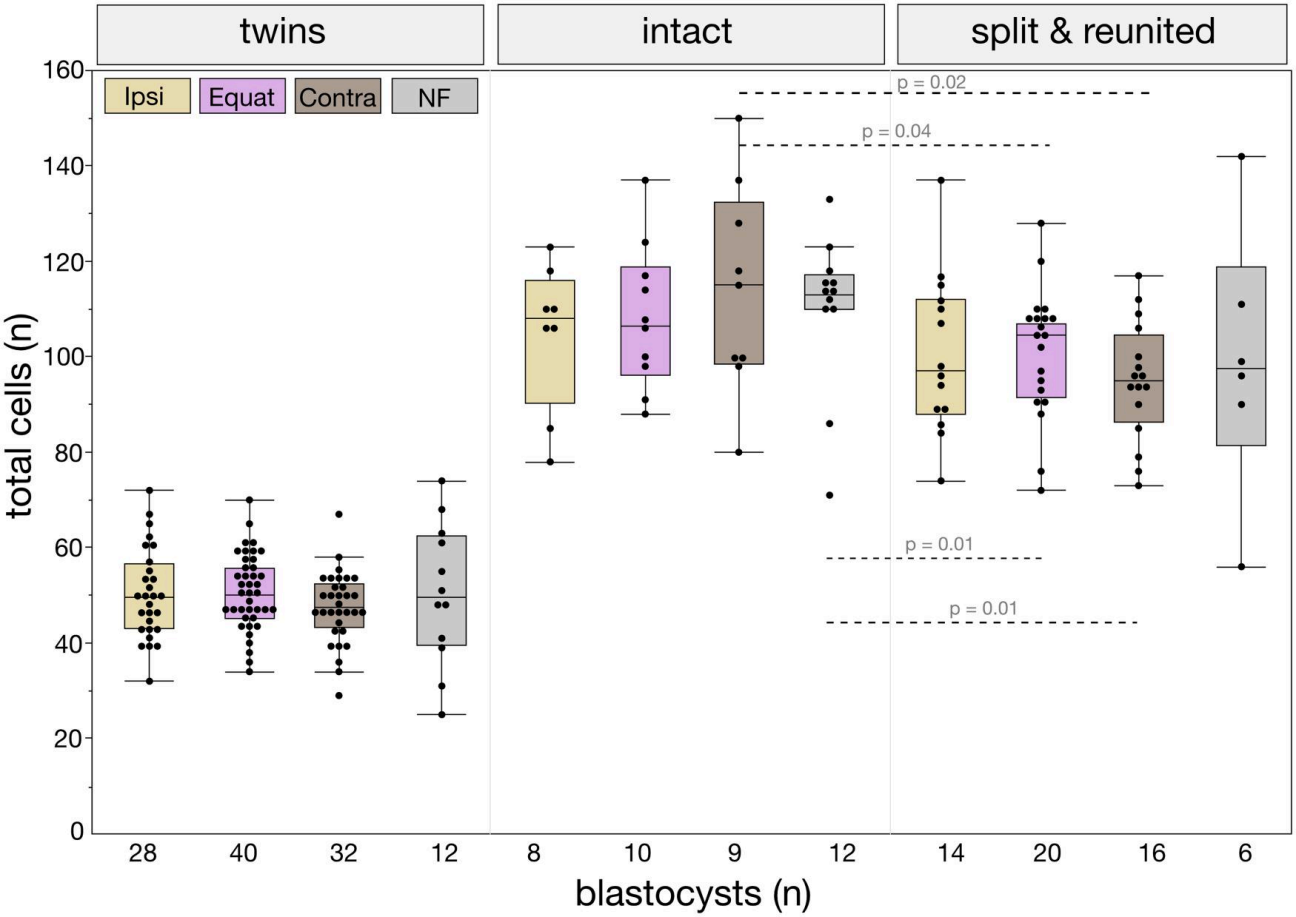
**Embryonic cell proliferation is preserved after ICSI and splitting.** Total cell numbers of blastocysts were counted after staining with Hoechst 33342. The horizontal lines of the box are Q1, median and Q3; the whiskers that extend from the box correspond to the range defined as 1.5 × IQR (interquartile range) from Q1 and Q3. The dots on the box plots represent individual embryos. Total cell counts were similar within twins, intact, and split-reunited embryos, irrespective of the means of fertilization (ICSI vs natural fertilization, NF), whereas cell counts were different across intact and split-reunited embryos (Wilcoxon test). Ipsi, ipsilateral = animal (A) pole; equat, equatorial; contra, contralateral = vegetal (V) pole.

Given the aim of the study, one prerequisite had to be satisfied in order to proceed with the epiblast analysis: the total cell number of the blastocyst should not be affected by the ICSI, otherwise the differences in the epiblast formation that we want to investigate could be due to trivial differences in the total cell number between the ICSI and NF. Clearly, if there were more cells in total, there would also probably be more epiblast cells. Conversely, if there were fewer total cells in total (a trait characteristic of aneuploid embryos, Bolton *et al.*, 2016), there would also probably be fewer epiblast cells. Therefore, we applied two controls (Fig. 2; Supplementary Table S2) to assure that our ICSI and splitting procedure, although involving substantial physical manipulation, would not, *per se*, compromise the total cell numbers of twin and cotwin blastocysts. Firstly, we included a group of NF oocytes, which served as a control for the effect of the invasive microinjection. The blastocyst rate was lower after the ICSI (40.3 ± 19.0%) than after NF (75.9 ± 13.6%), which is not surprising given the difference in manipulation. As a second control, a subset of the split blastomeres was reunited to reconstruct the original 2-cell embryos, expecting that the reconstructed embryos would not only yield the same blastocyst rates as non-split counterparts, but also have the same total number of cells as that of non-split embryos. The total cells of the twin and control embryos were counted at the blastocyst stage (96 h) after staining the nuclear DNA with Hoechst 33342. The total cell counts of each single embryo are provided in Supplementary Table S2. Within each manipulation (twins, intact, split, and reunited) ICSI blastocysts had the same number of total cells as those obtained by NF, and the total cell numbers were similar across the ICSI sites. After splitting, the twins had total cell counts that were half of the intact controls (≈ 50 vs ≈ 100, Fig. 2).

Taken together, the results obtained support the validity of using our ICSI model to study the effects of the fertilization site on the epiblast imbalance in mouse embryogenesis. While ipsilateral ICSI may pose a risk of spindle damage and consequent aneuploidy, this risk is not corroborated by cell count analysis. The matter will be evaluated further in a subsequent analysis.

### Twin–cotwin imbalance in gene expression is more likely to exist at the protein level than at the transcript level

If a blastocyst’s epiblast imbalance depended on the oocyte’s fertilization imbalance, then changing the site of sperm deposition in the oocyte—via ICSI—should change the extent of the epiblast imbalance. Since the genes that instruct the epiblast and the other lineages are not expressed in the zygote, detecting their transcripts at later stages is an indication that the instructive processes are active. Thus, a transcriptome analysis would allow us to capture the expression of several relevant genes at once. To test the epiblast imbalance, we retrieved the blastocysts’ gene expression data from our previously published dataset (GSE241089), which included 42 pairs of twin blastocysts (14 each from ipsilateral, equatorial, or contralateral ICSI), sampled from a larger group of 102 (3×34) pairs. In order to be used, the blastocyst subset of data required reprocessing of the raw data (GSE241089), which led the depth to increase from 7660 (Nolte *et al.*, 2025) to 19 341 genes (present study). The reason for this increase is that embryonic genes in blastocysts, being at the beginning of their expression, are expressed at low levels and were excluded from our previous study focused on the 2-cell stage (Nolte *et al.*, 2025). We compared twin and cotwin for the transcript levels of genes that are characteristically expressed in the blastocyst’s cell lineages (Azami *et al.*, 2017; Franzén *et al.*, 2019; Allegre *et al.*, 2022; Suzuki *et al.*, 2022; Weberling *et al.*, 2025) and we also included housekeeping genes as a reference (Mamo *et al.*, 2007; Jeong *et al.*, 2014). Some of the 42 embryos were discarded as a result of pipeline and quality check criteria (Materials and Methods). Moreover, when a twin is discarded, then the cotwin must also be discarded. As a result, we were left with 7, 6, and 6 complete pairs in the ipsilateral, equatorial, and contralateral group, respectively. These samples encompassed 10 773 protein-coding genes. A summary of the whole RNA-seq data (TPM values) is provided in Supplementary Table S3. The following epiblast genes were found expressed in our RNA-seq dataset: *Cdh1*, *Cldn6*, *Cldn7*, *Cripto*, *Epcam*, *Klf4*, *Nanog*, *Pou5f1*, *Sox2*, *Stat3*, *Tfcp2l1*, and *Zfp42*. The following primitive endoderm genes were found expressed: *Dab2*, *Esrrb*, *Fgfr2*, *Gata6*, *Kdelr3*, *Klf5*, *Lama1*, *Lrp2*, *Pdgfra*, *Sox17*, and *Timd2*. The following trophectoderm genes were found expressed: *Cdx2*, *Elf5*, *Eomes*, *Gata3*, *Id2*, *Klf5*, *Phlda2*, *Plac1*, *Tead4*, *Tfap2c*. The following housekeeping genes were found expressed: *Actb*, *Eef1e1*, *Gapdh*, *H2az1*, *Hprt1*, *Pgk1*, *Ppia*, *Tbp*, *Tubb4b*, and *Ubc*. A summary of the RNA-seq data of the cell lineage and housekeeping genes is provided in Supplementary Table S4. Since a dataset of thousands of gene transcripts can be queried to answer a variety of other questions besides cell lineage allocation, we leveraged it to revisit a possibility that ICSI could cause aneuploidy; in turn, aneuploidy could result in differential gene expression. The results, summarized in Supplementary Fig. S1, compare the expression levels of genes involved in mitotic chromosome stability between intact blastocysts after equatorial ICSI (set to 1) and intact blastocysts after ipsilateral and contralateral ICSI. Results show that it is not so much ipsilateral ICSI as contralateral ICSI that causes a reduction of ∼50% in the mRNA levels of relevant genes. This contrasts with cell counts though, which are unaffected, reminding us of post-transcriptional regulation. We will return to this point at the end of the next paragraph.

**Figure 3.**
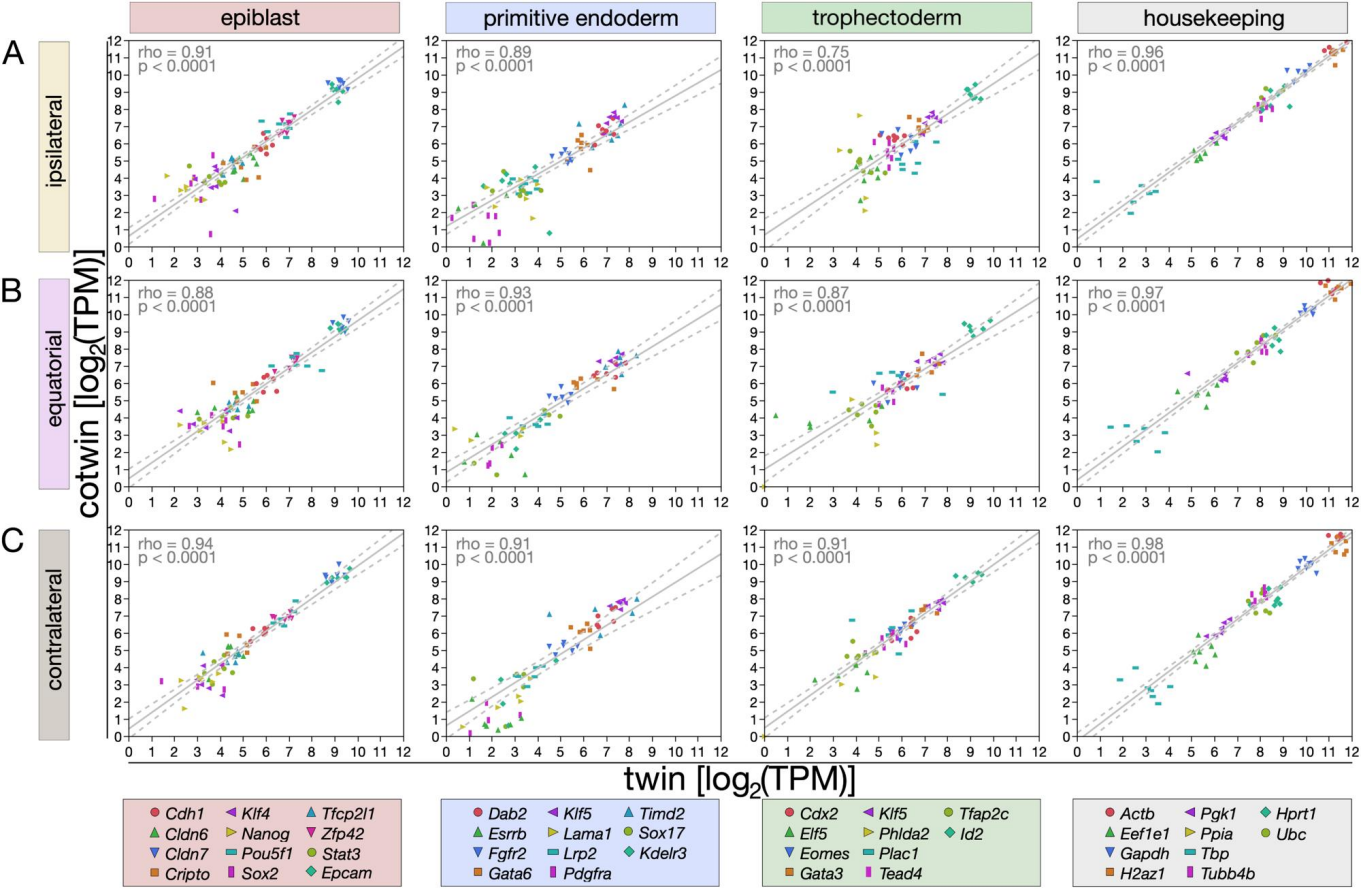
**Supervised transcript analysis of cell lineage genes does not capture the epiblast imbalance.** Genes characteristically expressed in each cell lineage were quantified by RNA-seq transcripts per million (TPM) in twin and cotwin blastocysts of the ipsilateral (**A**), equatorial (**B**), and contralateral (**C**) ICSI (N = 7, 6, 6 pairs, respectively). Note: ‘ipsilateral’ = animal (A) pole, ‘contralateral’ = vegetal (V) pole. Housekeeping genes were included as a reference. Paired logarithmic values on base 2 of the TPM values were rendered graphically as *X*–*Y* scatter plots. To assign twin and cotwin consistently to the *X* or *Y* axis, in each pair the twin blastocyst (‘a’) was defined as the one with lower level of *Actb* mRNA (fewer cells) and placed on *X*, the cotwin blastocyst (‘b’) was defined as the one with higher level of *Actb* mRNA (more cells) and placed on *Y*. The linear correlation of twin and cotwin blastocysts’ log_2_(TPM values) was calculated with Spearman’s ρ (rho).

**Figure 4.**
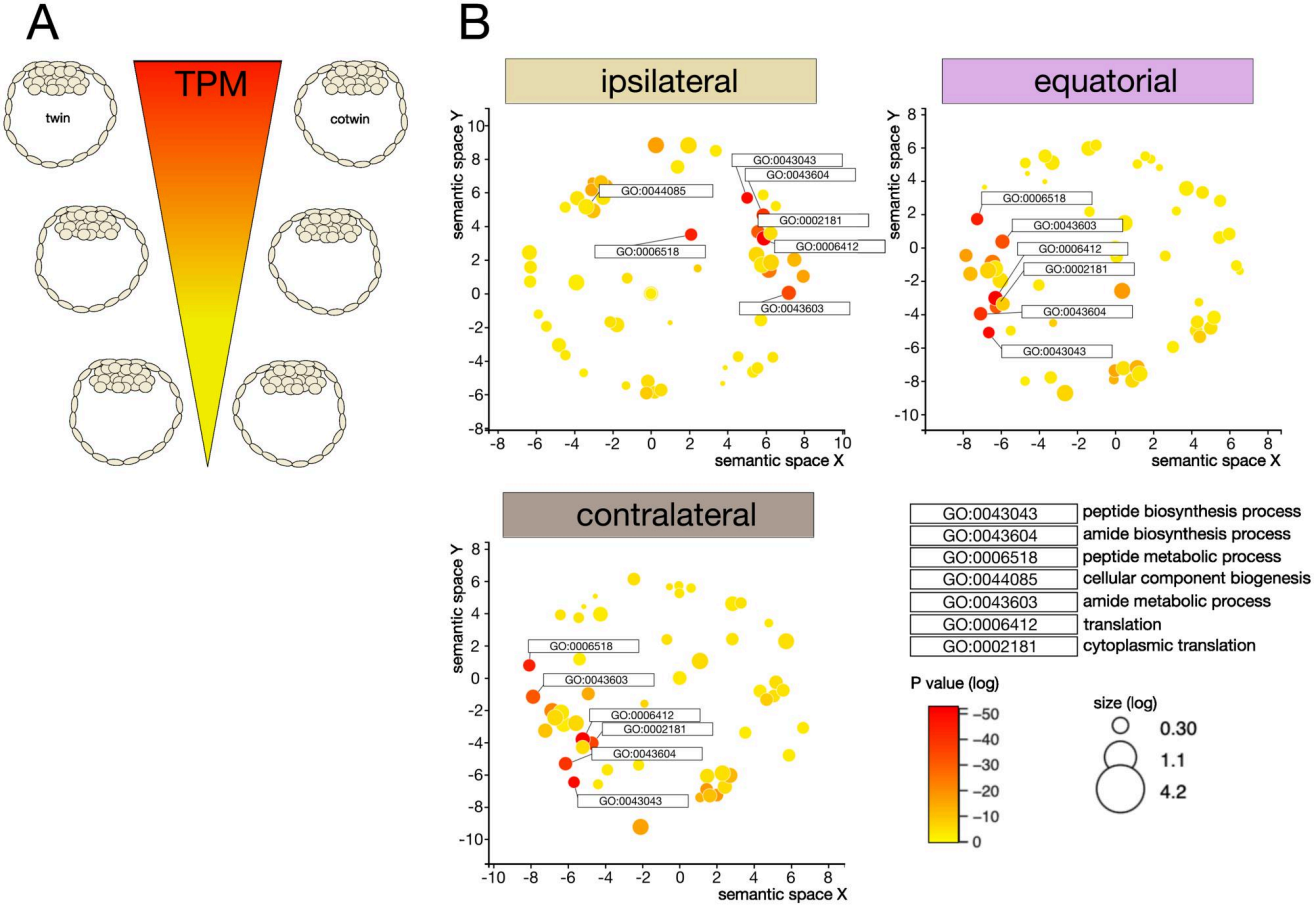
**Unsupervised transcript analysis of all detected genes hints at a post-transcriptional origin of the twin–cotwin imbalances.** (**A**) Absolute transcripts per million (TPM) differences of twin and cotwin were sorted in descending order in each group (ipsilateral, equatorial, contralateral ICSI). (**B**) Sorted lists were then subjected to gene ontology (GO) analysis in the biological process using REVIGO (Supek *et al*., 2011). The bubble plots show the cluster representatives (i.e. terms remaining after the redundancy reduction) of the inter-blastocyst differences (TPM), in the ontology ‘biological process’, in a two-dimensional space derived by applying multidimensional scaling to a matrix of the GO term semantic similarities. Plots were generated using REVIGO (Supek *et al*., 2011).

We generated *X*–*Y* scatter plots of the TPM values of twin and cotwin for each gene family (epiblast, primitive endoderm, trophectoderm, housekeeping) in an initial approach. We call this approach ‘supervised’ because we imposed conditions, such as assigning the expression values of selected genes to twin and cotwin. We faced a long-standing problem (VerMilyea *et al.*, 2011) given the paired structure of the data (twin, cotwin): it is not known which member in a pair is ‘a’ and which ‘b,’ in order to assign them consistently to the axes of *X*–*Y* scatter plots (supervised approach). We solved this problem based on studies in which it was shown that preimplantation mouse embryos with more cells have higher levels of the housekeeping gene *Actb* mRNA (Giebelhaus *et al.*, 1983, 1985). We confirmed this relationship between the cell number and *Actb* mRNA in our dataset, leveraging the 2-fold difference in the total cell number between twins and intact (non-split) controls (Supplementary Fig. S2). Accordingly, we defined ‘twin (a)’ as the one with a lower level of *Actb* mRNA (fewer cells) and ‘cotwin (b)’ as the companion with a higher level of *Actb* mRNA (more cells). The twin–cotwin linear correlation of TPM data was always positive and close to 1 (Fig. 3), which does not support an imbalance of epiblast production depending on the site of fertilization. This may mean that differences of a few cells among the total cells are below the sensitivity of our RNA-seq, or that the differences emerge at the post-transcriptional level. To try to resolve these possibilities, we designed a different type of analysis, taking into account all protein-coding genes, not just those of the cell lineages. Volcano plots were used to compare twins and cotwins for all 10 773 protein-coding mRNAs detected in this study. The lesson turned out to be different from that of the scatter plots: while a few genes (≈ 100) were expressed differently between twins and cotwins, those genes were distinct among the three groups (Supplementary Fig. S3). The group size of ≈ 100 did not support a meaningful GO analysis. We, therefore, changed our approach, abandoning supervised analysis in favor of unsupervised analysis, in which TPM differences were examined regardless of gene families and a–b ranking. Absolute TPM differences of twin and cotwin were sorted in descending order in each group (ipsilateral, equatorial, and contralateral ICSI). Sorted lists were then subjected to GO analysis in the biological process using REVIGO (Supek *et al.*, 2011). The result was a recurrent prevalence of GO-biological process terms related to ribosomes and protein synthesis in all three groups (Fig. 4).

Taken together, these data suggest that RNA-seq is not adequate to expose the twin–cotwin differences, probably because the latter differences emerge or increase downstream of the transcripts when these are translated to proteins.

### Analysis of protein markers substantiates that the extent of the epiblast imbalance depends on the fertilization site

Given the outcome of the transcript-based GO analysis, suggesting that the differences between twins and cotwins are at the protein level, we used the remaining 60 of 102 pairs of twin blastocysts (20 from each ICSI site) to perform protein analysis, counting the epiblast cells of twin and cotwin after triple immunofluorescence for NANOG, SOX17, and CDX2 (Schwarzer *et al.*, 2012; Casser *et al.*, 2017), which are crucial regulators of the cell lineages. Accordingly, we simultaneously obtained not only the counts of epiblast but also those of the primitive endoderm and trophectoderm. Fourteen, 20, and 16 pairs of the 20 pairs of each ICSI group were successfully recorded after imaging in the ipsilateral, equatorial, and contralateral group, respectively (Supplementary Table S5). The signal was specific (Supplementary Fig. S4), and it is reassuring that the minimum viable number of four epiblast cells (Morris *et al.*, 2012) was attained in all groups (contralateral twin 5.0 ± 2.5; contralateral cotwin 5.4 ± 2.0; equatorial twin 4.4 ± 2.3; equatorial cotwin 5.0 ± 1.8; ipsilateral twin 4.9 ± 2.6; ipsilateral cotwin 5.7 ± 2.1; mean ± SD; *P* > 0.0719, Wilcoxon test; Supplementary Table S5).

**Figure 5.**
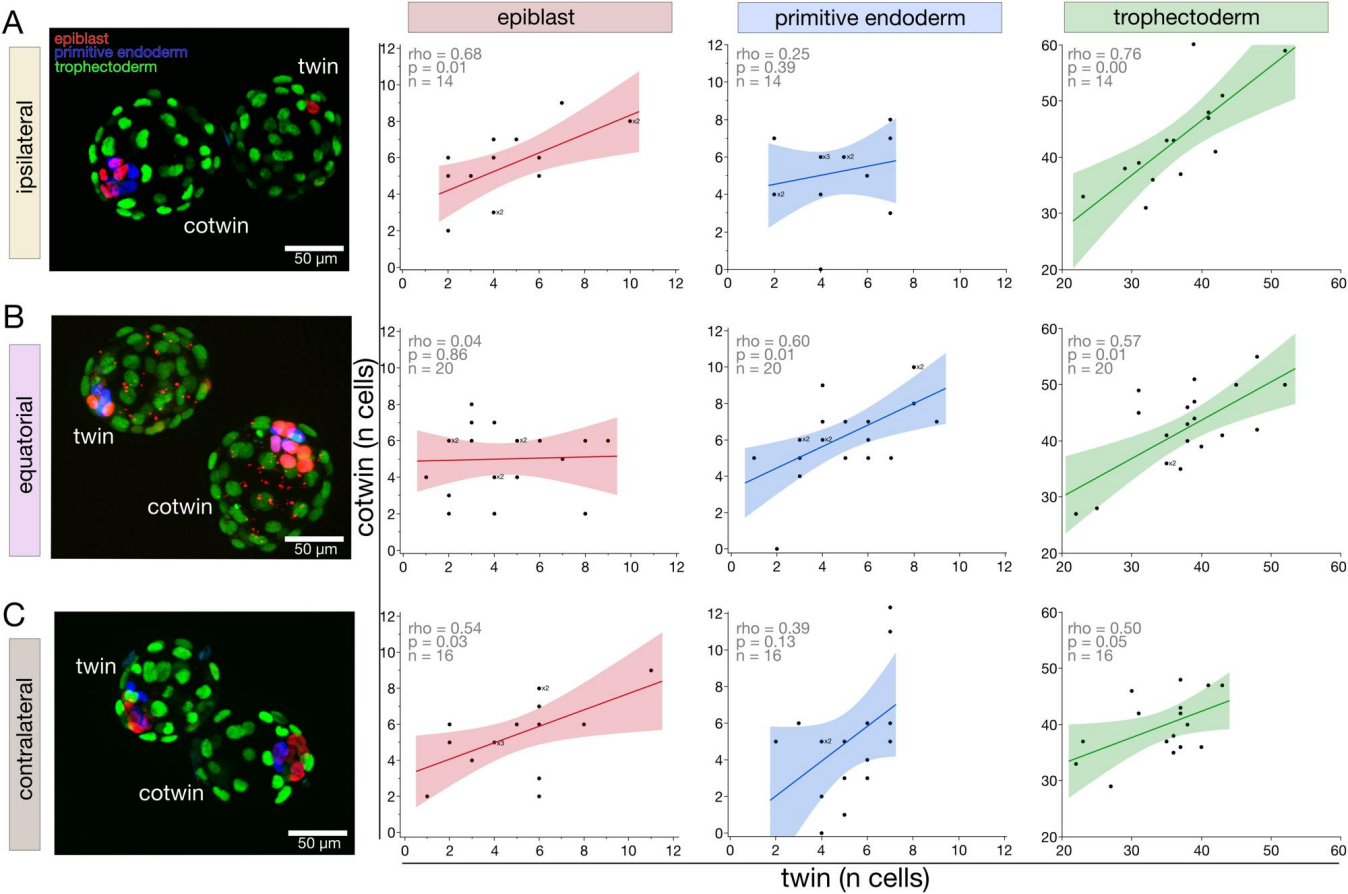
**Epiblast imbalance is more pronounced after equatorial ICSI.** Representative images of immunostained twin pairs after ipsilateral (**A**), equatorial (**B**), and contralateral (**C**) ICSI. Note: ‘ipsilateral’ = animal (A) pole, ‘contralateral’ = vegetal (V) pole. The cells assigned to each lineage were counted (NANOG-positive epiblast, red; SOX17-positive primitive endoderm, blue; CDX2-positive trophectoderm, green) in each twin pair of each ICSI group (ipsilateral, equatorial, contralateral). Counts were rendered graphically as scatter plots (*X* axis, cells counted in twin ‘a’; *Y* axis, cells counted in cotwin ‘b’). In some cases, the counts of two or three different embryos were identical and thus were superimposed in the scatter plot, in which case ×2 or ×3 was written next to the dot. The linear correlation of twin and cotwin blastocysts was calculated with Spearman’s ρ (rho).

We generated *X*–*Y* scatter plots of the cell numbers of twin and cotwin for each ICSI site and each cell lineage. Given the paired structure of the data (twin, cotwin), we again faced the same problem as during the transcriptome analysis: it is not known which member in a pair is ‘a’ and which ‘b,’ in order to assign them consistently to the axes of *X*–*Y* scatter plots. We defined ‘twin (a)’ as the one with a lower total cell number and ‘cotwin (b)’ as the companion with a higher total cell number. The cell counts of each single embryo in the cell lineages are provided in Supplementary Table S5. While the a–b linear correlation was positive across all lineages and ICSI sites (Fig. 5A–C), there was a slope of zero only in the case of the epiblast after equatorial fertilization (Fig. 5B). Note that the counts of the three cell lineages originated from the same embryos within each ICSI site: this allows one to exclude that the peculiar epiblast behavior (Fig. 5B) was due to a few defective embryos in the pool, as this should have spoiled the correlations of the other two lineages as well, but it did not.

**Figure 6.**
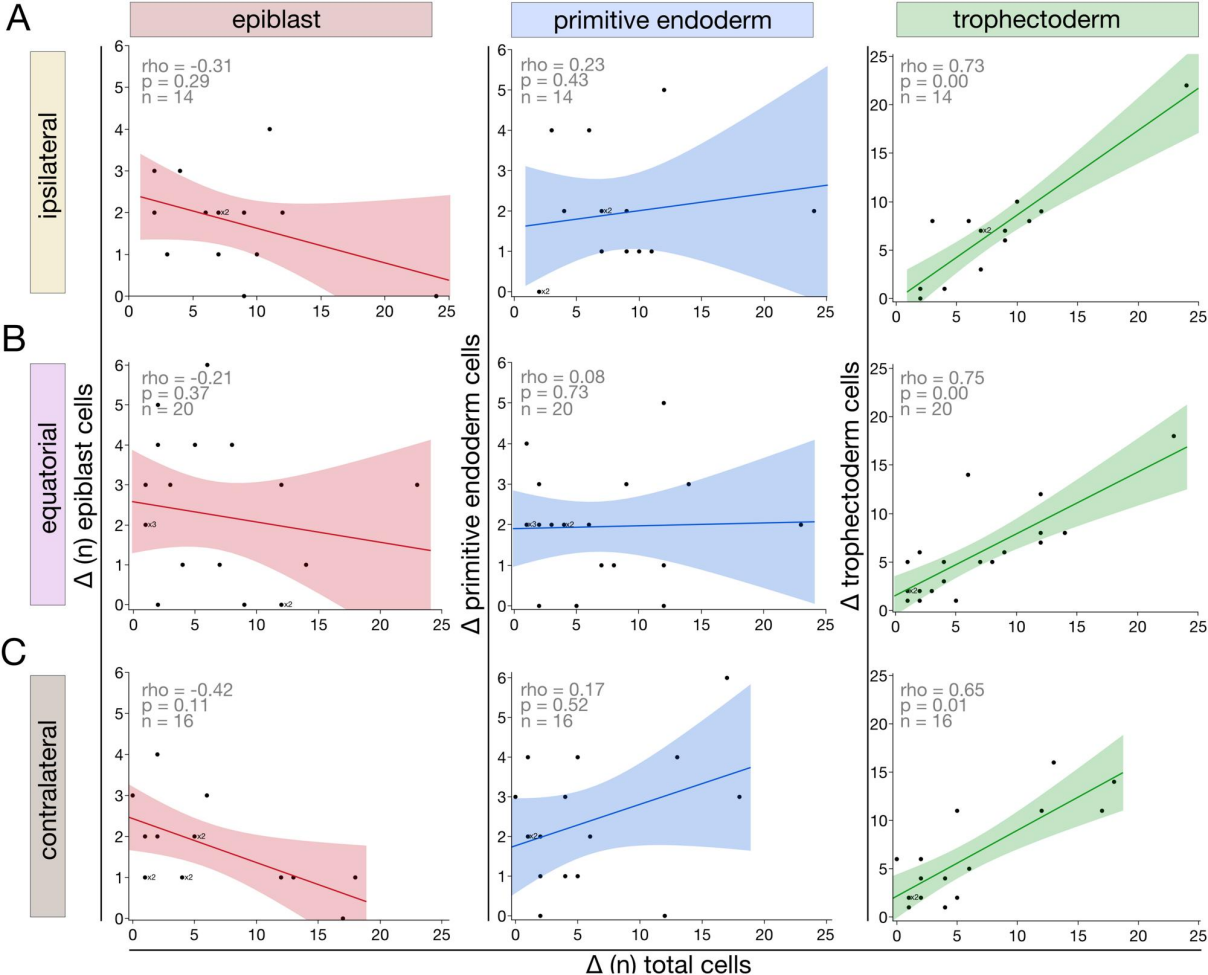
**Epiblast imbalance is not explained by differential cell proliferation of the sister blastomeres.** In principle, the more cell mitotic divisions follow each other, the more asynchronous the mitotic clones become, and, thus, cell number differences can ensue. To assess this possibility, the absolute difference (Δ) between cells in the same cell lineage (epiblast, primitive endoderm, trophectoderm) was calculated and plotted against the Δ in the total cells, for each twin pair of each ICSI group (**A**, ipsilateral; **B**, equatorial; **C**, contralateral). Note: ‘ipsilateral‘= animal (A) pole, ‘contralateral‘= vegetal (V) pole. In some cases, the Δ of two or three different embryos were identical and thus were superimposed in the scatter plot, in which case x2 or x3 was written next to the dot. This approach allowed us to conclude that the epiblast imbalance is not a trivial manifestation of total cell imbalance. The linear correlation of twin and cotwin blastocysts was calculated with Spearman’s ρ (rho).

Given the method above used to construct the scatter plots, i.e. assigning the blastocyst with fewer cells as ‘a’ and that with more cells as ‘b’, it created a possibility that the correlations varied for the following reason: one blastomere produced more epiblasts simply because it produced more cells in total. It is well known that one 2-cell blastomere often enters mitosis before the other, which can influence the cell number accumulation. We refuted this possibility as follows. We calculated the absolute differences in cell counts (Δ), and plotted the Δ of each cell lineage against the Δ of the total cell numbers (Fig. 6; Supplementary Table S6). Results show that there is a Δ of two to three cells in the epiblast compartment even when the total cell numbers are the same (Δ = 0), irrespective of the ICSI being ipsilateral (Fig. 6A), equatorial (Fig. 6B), or contralateral (Fig. 6C). Moreover, the epiblast behaves distinctly from the other two cell lineages: There is a negative relationship with total cell number only in the epiblast.

Taken together, these protein marker data support the centerpiece of our study, namely, that the epiblast imbalance is not a generic feature of 2-cell stage blastomeres, but responds to the region where the oocyte was fertilized and is more pronounced at the equator. Moreover, the imbalance is intrinsic rather than a timing delay, because there is a persistent slight imbalance of the epiblast (∼2–3 cells on average) even in the most balanced cases of total cell number.

### Epiblast imbalance reaches beyond the blastocyst stage

The analyses of mRNA and protein markers of the previous sections were destructive of the embryos, therefore, they precluded an obvious follow-up, which was to check whether the differences in the epiblast compartment would extend past the blastocyst stage. In order to obviate this limitation, we adopted the ES cells paradigm, in which each cell of the inner cell mass cell (including the epiblast) is converted into a colony of pluripotent ES cells that divide indefinitely. We took advantage of the Oct4-GFP transgene as a live-cell marker of pluripotency to count the colonies without using destructive methods (Boiani *et al.*, 2002). Assessing the quality of the ES cells was beyond the scope of this analysis.

**Figure 7.**
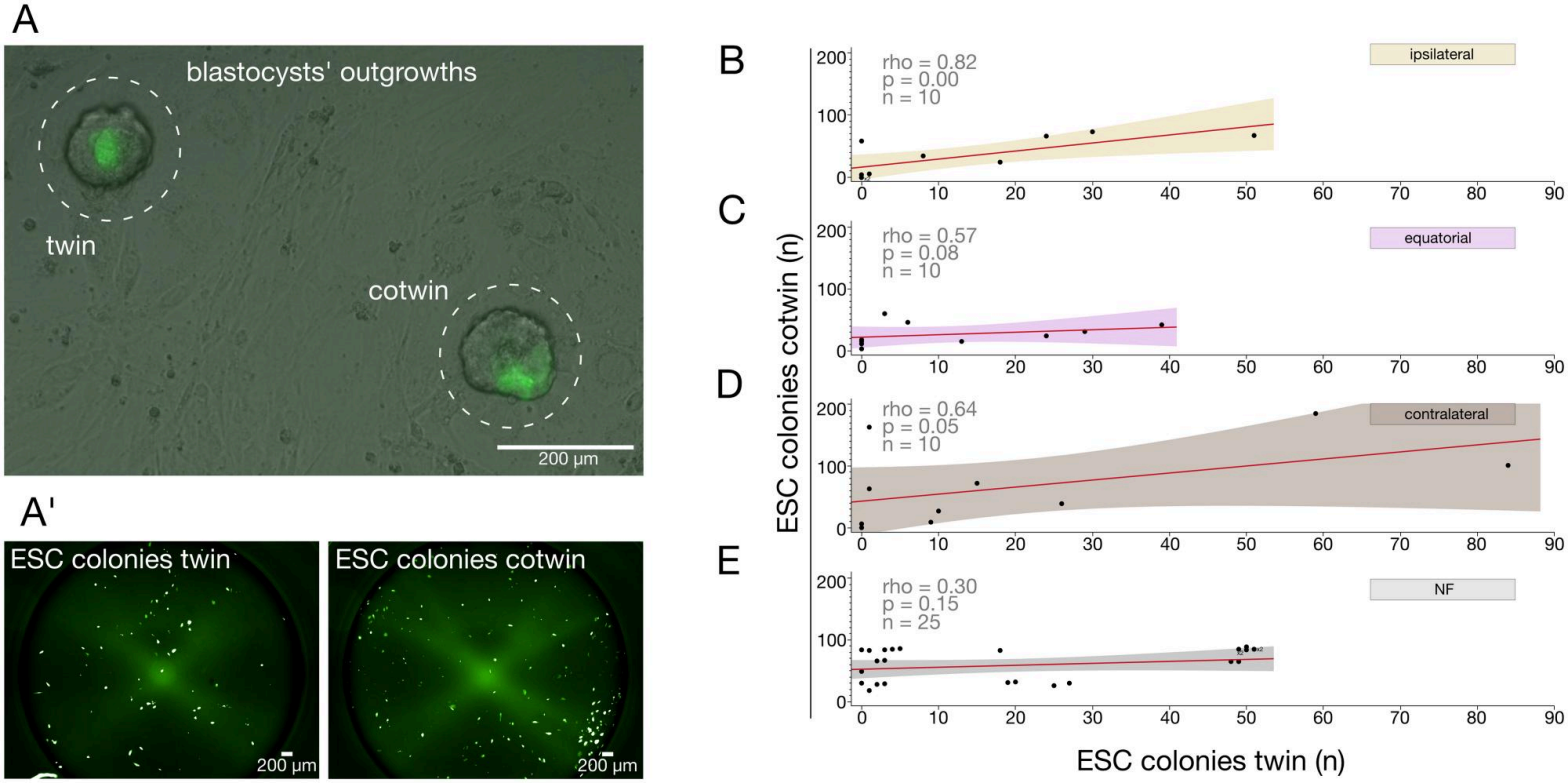
**Derivation of ES cells (ESC) from twin blastocysts as a functional assessment of the imbalance in the epiblast cell number.** (**A**) After individual blastocysts (B6C3F1×OG2) had formed outgrowths and these were dissociated into single cells, each epiblast cell gave rise to a colony of ES cells. (**A’**) Numbers of colonies were counted using an Operetta high-content imager. (**B–E**). Paired ES cell colony counts of the twin blastocysts from the three ICSI sites (**B**, contralateral, N = 10 pairs; **C**, ipsilateral, N = 10 pairs; **D**, equatorial, N = 10 pairs) were rendered graphically as *X*–*Y* scatter plots (*X* axis, ES cell colonies formed by twin ‘a’; *Y* axis, ES cell colonies formed by cotwin ‘b’), compared to the scatter plots of twins derived from NF embryos (**E,** N = 25 pairs). Note: ‘ipsilateral’ = fertilization at the animal (A) pole, ‘contralateral’ = fertilization at the vegetal (V) pole. In some cases, the counts of two or three different embryos were identical and thus were superimposed in the scatter plot, in which case x2 or x3 was written next to the dot. The linear correlation of twin and cotwin embryos calculated with Spearman’s ρ (rho).

We used spermatozoa of the OG2 mouse strain, which carries the Oct4-GFP transgene, to produce 150 additional zygotes via ICSI (⅓ ipsilateral, ⅓ equatorial, and ⅓ contralateral), and split them at the 2-cell stage as described. Fifty NF zygotes served as a control. Instead of lysing the twins for transcriptome analysis or fixing them to count the cells at the blastocyst stage, we plated the blastocysts on the feeder layer to allow for outgrowth formation and ES cell derivation as per our established procedure (Fig. 7A). Pluripotent colonies were visualized by Oct4-GFP fluorescence and counted using an Operetta high-content imager (Fig. 7A), keeping track of the original pair associations and the site of the ICSI (Supplementary Table S7).

The overall efficiency of the ES cell derivation in our hands was ≈70% (number of blastocysts that yielded ES cell colonies/number of blastocysts plated on feeders) and was uniform among both ICSI and NF twin pairs. However, the twin–cotwin relationship varied depending on the ICSI site. We drew *X*–*Y* scatter plots of the ES cell colony numbers of the twin and cotwin for each ICSI site and calculated the linear correlations. The correlations of ES cell numbers between twin and cotwin reflect those previously described for the epiblast, with higher linear correlation after contralateral and ipsilateral ICSI (Fig. 7B and D) and lower correlation for equatorial ICSI (Fig. 7C). It may also be noted that the ES cell correlation between twin and cotwin in the case of equatorial ICSI was closer to that of the NF ones (Spearman’s ρ 0.57 vs 0.30; Fig. 7E), consistent with the notion that natural fertilization occurs predominantly in the animal hemisphere near the equator.

Based on these results, we conclude that the twin epiblast imbalance persists beyond the blastocyst stage, particularly after equatorial fertilization.

### Variable partition of the non-homogeneous ooplasm sets the stage for the epiblast imbalance

If the epiblast imbalance depended on the oocyte, then the ooplasm should be non-homogeneous, with differences in composition between regions, for example, hemispheres, and these differences should be apportioned variably when the first zygotic division takes place. Two obstacles lie in the way of testing these predictions: (1) When zygotes divide in a culture plate laid in an incubator, it is almost impossible to reconstruct the relationship between the cleavage and A–V axis the next day, because the oocytes have rolled and rotated during the time from fertilization to the first cleavage; (2) regional differences in oocyte composition require a proteome analysis of the hemispheres of individual oocytes, but the bisection of oocytes is mastered by few researchers, and proteomic analysis of half-oocytes by even fewer. We devised solutions to overcome these obstacles as follows.

**Figure 8.**
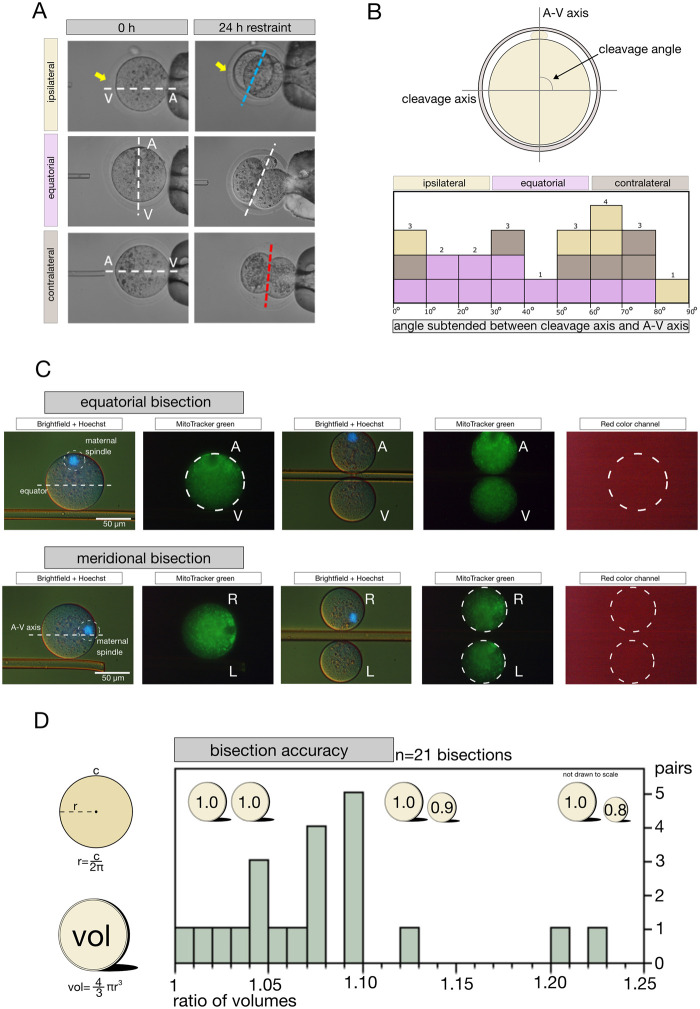
**Micromanipulation approaches used to test for variable partition of non-homogeneous ooplasm.** (**A**) Immobilization of oocytes to measure angles of first cleavage after site-specific ICSI. One oocyte was injected at a time with a single sperm head. After injection, the oocyte was not released, but held in position for 24 h, thereby allowing for the first mitotic cleavage to take place in the micromanipulation chamber. Representative images of oocytes of each ICSI group, showing the A–V axis of oocytes, the cleavage axis, and the hole in the ZP (yellow arrow). Scale bar, 10 µm. Note: ‘ipsilateral’ = animal (A) pole, ‘contralateral’ = vegetal (V) pole. (**B**) Distribution of angles subtended between the A–V axis and the first cleavage axis of 22 zygotes. Note that while the ipsilateral and contralateral ICSI populated the high end of the angle distribution (60–90°), the equatorial ICSI was distributed more evenly across angles. (**C**) Bisection of oocytes for subsequent proteome analysis. After staining with Hoechst 33342 and MitoTracker Green to visualize the spindle and the whole ooplasm, MII oocytes were oriented so as to have the spindle at the 12 o’clock position in case of equatorial bisection, or 3 o’clock position in case of meridional bisection. An additional picture was taken also in the red color channel, to confirm the specificity of the MitoTracker Green staining. Bisection was conducted using a borosilicate capillary (outer diameter 15 µm at the tip), pressing it downward in the middle to divide the oocyte into two parts of approximately equal volume corresponding to the A and V (Video 1) or to the L and R (Video 2) hemispheres, relative to the spindle position. (**D**) Accuracy of bisection, i.e. the ability to produce two halves of equal volume, was evaluated. The circumferences (c) of the halves were measured using Fiji, the radius (r) was extracted from the formula c = 2πr, and used to calculate the volume (V) using the formula V = 4/3πr^3^. The volumes of the two sister hemispheres were then divided by each other (larger volume/smaller volume). A perfect bisection should produce a ratio of 1. Histogram shows the distributions of the actual ratios, with an average of 1.079 and a SD of 0.055, meaning that the volumes differ on average by 7.9±5.5%. A, animal; V, vegetal; A–V, animal–vegetal axis; L, left (hemisphere) relative to A–V axis; R, right (hemisphere) relative to A–V axis.

We secured an invariant view of the oocytes and their first cleavage by holding the sperm-injected oocytes physically immobilized, one at a time, in the micromanipulation chamber for 24 h, as described (Nolte *et al.*, 2025). Pictures were taken twice, firstly, at the time of ICSI and then 24 h later at the early 2-cell stage (Fig. 8A). The unchanged position of the ICSI hole in the zona pellucida and the dent left by the ICSI needle in the oolemma attest to the successful immobilization of the oocytes. We used the pictures to measure and analyze the angle subtended between the initial A–V axis of 22 individual oocytes and their cleavage axis (Supplementary Table S8). We observed that a contralateral or ipsilateral ICSI was followed more often (70% of those cases) by cleavage perpendicular to the A–V axis, while an equatorial ICSI was followed by division at any angle (Fig. 8B). These are just correlations, yet, they support that the diversity of the two blastomeres in terms of epiblast production is related to the variability in the angles of first cleavage.

Variability in the angles of first cleavage is not sufficient to confer differences on the blastomeres unless the ooplasm is also non-homogeneous in composition. One would need to compare the molecular profiles of different regions of the same oocytes to determine whether this is the case; some molecules should be more abundant in one region than another. Since the protein analysis was the one that previously revealed the epiblast imbalance of the twin blastocysts, while the transcript analysis did not, we chose to examine the oocyte regions by protein analysis. Therefore, we manually bisected MII oocytes along the equator (equatorial bisection; Video 1) or along the A–V axis (meridional bisection; Video 2) to reproducibly obtain predefined regions. To have reference points for the bisection, we co-stained the oocytes with Hoechst 33342 and MitoTracker Green in order to visualize, respectively, the spindle and the global volume of the ooplasm (Fig. 8C). While the bisection cuts the cell, it does not cut the spindle, which remains associated with one of the two halves. In the case of equatorial bisection, it was clear which hemisphere was which, given the definition of A hemisphere as the one that contains the spindle (as opposed to the V hemisphere lacking the spindle; Video 1). In the case of meridional bisection, it is not given to know which hemisphere is ‘right’ (R) and which is ‘left’ (L) relative to the A–V axis, and upon bisection, the spindle can end up in either hemisphere. We arbitrarily—but consistently—called ‘R’ the hemisphere with the spindle and ‘L’ the one without the spindle (Fig. 8C; Video 2). The accuracy of our bisection was high. It yielded two halves almost equal in volume, as indicated by the statistical distribution of the volume ratios of the hemispheres of 21 oocytes bisected for training purposes (Fig. 8D). If the bisection were perfect, the ratio would be 1; we obtained a modal ratio of 1.1 (Fig. 8D), indicating that the difference in volume between the halves is ≈ 10% (precisely 7.9 ± 5.5%).

**Figure 9.**
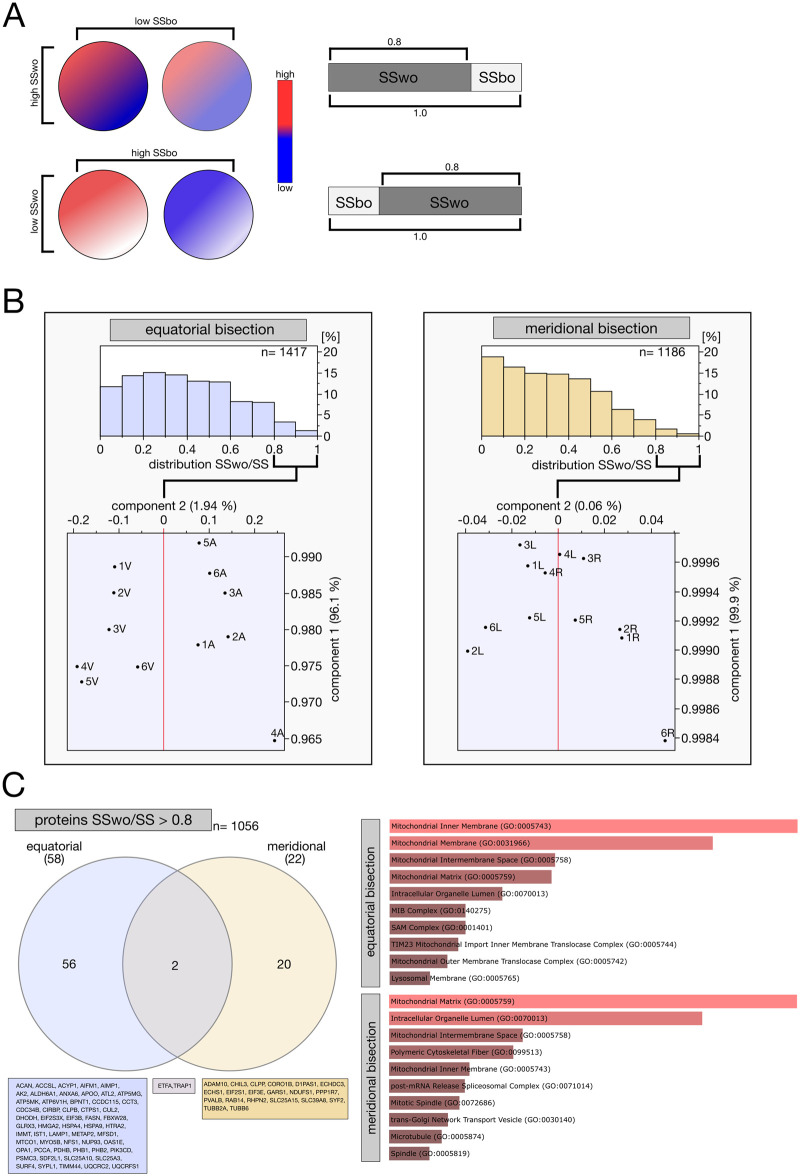
**Assessment of protein regionalization (ooplasmic non-homogeneity) by means of proteome analysis conducted on oocyte hemispheres.** (**A**) Conceptual illustration of gene expression variance (total sum of squares, SS) partitioned into SS within-oocyte (SSwo) and SS between-oocyte (SSbo). The upper panel illustrates the case of high SSwo with low Ssbo (protein heterogeneity is dominant within oocytes), while the lower panel illustrates the case of low SSwo with high SSbo (protein heterogeneity is dominant between oocytes). Colors indicate relative expression levels. (**B**) The statistical distributions of SSwo/SS ratios of proteins are different after equatorial versus meridional bisection (Spearman’s ρ = 0.1630, *P* < 0.0001). Principal component analysis shows that A and V hemispheres from equatorial bisection are resolved when considering proteins with SSwo/SS >0.8, while this is not the case when considering the L and R hemispheres from meridional bisection. (**C**) Proteins were ranked by descending SSwo/SS ratio and those with SSwo/SS > 0.8 (n = 58 after equatorial bisection, n = 22 after meridional bisection) were compared by Venn diagram. Overlap is minimal (n = 2) indicating that distinct sets of proteins are regionalized along the A–V axis vs equator. The identities of the proteins are shown in the boxes underneath the Venn diagram. Bars show the results of overrepresentation analysis of the 78 unique proteins (78 = 58 + 22 − 2) proteins using *Enrichr* (Chen *et al*., 2013; Kuleshov *et al*., 2016; Xie *et al*., 2021). A, animal (hemisphere); V, vegetal (hemisphere); L, left (hemisphere) relative to A–V axis; R, right (hemisphere) relative to A–V axis.

We were interested in the protein composition of hemispheres and systematic differences between hemispheres (Fig. 9). Therefore, we bisected an additional 12 oocytes, 6 of them along the equator (equatorial bisection) and 6 along the A–V axis (meridional bisection), for a total of 24 halves for analysis by mass spectrometry. The number may seem low, but it should be noted that this is the first application of proteomics to half mammalian oocytes, and that the halves we are going to compare originate from the same oocyte, which eliminates all confounders except one—the accuracy of the bisection (≈ 10%). Since this bisection error will influence the protein measurements, we anticipate that it will not be sufficient to look for quantitative differences that are consistently present across all pairs of hemispheres, but another statistical approach will have to be sought (see below). The 24 hemispheres were processed for mass spectrometry according to an innovative one-step microvolume method in which trypsin-digested cell lysates are injected directly into the mass spectrometer, thereby minimizing losses of material (Materials and Methods). A total of 2257 and 1837 proteins were detected after equatorial and meridional bisection, respectively. The proteomic datasets including the raw data are available in the PRIDE repository (http://proteomecentral.proteomexchange.org), with the dataset identifier PXD067319. A summary of the data is provided in Supplementary Table S9. Regarding the total number of proteins detected, 1417 were present in all hemispheres of the equatorial set, 1186 proteins were present in all hemispheres of the meridional set, and 1056 proteins were in common to both sets. Clearly, these numbers do not represent the whole proteome of oocytes, but they are, nonetheless, decent numbers considering that they were obtained from half an oocyte without any template amplification. We performed variance analyses of 1417 and 1186 protein levels, based on the premise that relevant patterns for this study are those where proteins (whichever may they be) have greater variability between hemispheres of the same oocytes than between the whole oocytes (Fig. 9A). This premise can be tested by analyzing the sum of squares (SS) of all items relative to the grand mean, and breaking it down into components, in each set (equatorial, meridional). To do so, one calculates the SS of all 12 hemispheres relative to their average, then the SS of the 6 oocytes (combined hemispheres) relative to their average (SS between oocytes, SSbo), and, finally, subtracts the SSbo from the SS to obtain the SS for the hemispheres within oocytes (SSwo). The ratio between the SSwo and the SS gives an estimate of how preponderant the differences between the hemispheres are, for example, ‘1’ means that differences within oocytes dominate, and ‘0’ means that differences between oocytes dominate. The statistical distributions of the SSwo/SS ratios are presented in Fig. 9B, showing that protein differences between hemispheres are more pronounced after equatorial than meridional bisection (the two distributions are different, Spearman’s ρ  =  0.1630, *P* < 0.0001). We applied PCA to the 1417 and 1186 proteins of the equatorial and meridional bisection, respectively, to identify any high-confidence differences between hemispheres. The A and V hemispheres in the equatorial bisection could not be resolved when all 1417 proteins were examined, but were resolved when proteins with SSwo/SS >0.80 (n = 58 proteins) were used for the PCA. By contrast, the L–R hemispheres in the equatorial bisection could not be resolved in any case (neither considering all 1186 proteins, nor those with SSwo/SS >0.80, n = 22) (Fig. 9B). Note that a ratio of SSwo/SS >0.80 means that the difference between the hemispheres is four times that between the oocytes, and four offers a wide margin of safety also with respect to the error introduced by the bisection (10% = 0.1). A Venn diagram analysis of the 58 and 22 proteins returned negligible overlap (n = 2), suggesting that the regional differences (A–V vs L–R) are robust (Fig. 9C). Overexpression analysis of the GO terms of the regionalized proteins using *Enrichr* (Chen *et al.*, 2013; Kuleshov *et al.*, 2016; Xie *et al.*, 2021) returned terms associated with mitochondria and endomembrane systems (Fig. 9C). How is it possible that two bisections perpendicular to each other produce the same GO effect? We recalled the MitoTracker staining (Fig. 8C), which showed that mitochondria are more abundant around the spindle. Since both bisections result in one hemisphere having the spindle and the other hemisphere lacking the spindle (our bisection cannot cut it in two but only displace it), this could explain why orthogonal bisections (equatorial, meridional) have the same mitochondrial GO signature.

Taken together, our results support that there are regions of different protein composition in the ooplasm. The proteins involved are few (78 among 1056; ≈ 7%), which should come as no surprise, because if there had been many proteins, colleagues would have realized already, using low-throughput methods such as immunofluorescence for candidate gene products. This non-homogeneity sets the stage for the effect of variable partition at first mitosis, which will eventually result in blastomeres producing different amounts of epiblast.

## Discussion

What this study has shown is that the progenies of the 2-cell stage blastomeres are imbalanced in epiblast production in a way that depends on the region where the oocyte was fertilized, and this difference can be increased or reduced under specific conditions, in mouse oocytes subjected to ICSI. Imbalance indicates a bias, rather than an absolute inability of one blastomere to form epiblast. Of the three regions tested, the ‘equatorial’, produced the largest epiblast imbalance at the blastocyst stage. By contrast, if the oocyte was fertilized at the V pole (something that does not usually happen in nature), the resulting two blastomeres were more similar to one another. Our data support a proposal that this positional effect may depend on how a regionally non-homogeneous ooplasm is apportioned into the blastomeres at the first zygotic division. This proposal is rooted in the historical model of Friedrich Seidel, which is known to few, since he published it more than 60 years ago in German not English (Seidel, 1952, 1960). We discuss the biological bases and implications of these observations in mice, and whether the mouse observations may be pertinent to human ICSI.

In order to illuminate the origin of the epiblast imbalance, our experimental strategy was to remove confounders that have been present in other studies. We harnessed the ICSI method in mice (Kimura and Yanagimachi, 1995) to rebalance fertilization by depositing the sperm head at the naturally unfavored V hemisphere of the oocyte, as well as in the A hemisphere hosting the spindle and in the cortex around the A–V midline (equator). Performing ICSI near the spindle is not recommended, due to the risk of perturbing the spindle and inducing embryonic aneuploidy. However, it appears that the risk of aneuploidy did not materialize in our study, based on the total number of cells, which should be reduced if the blastocysts were aneuploid (Bolton *et al.*, 2016). In addition to rebalancing the probability of the fertilization sites, the ICSI also allowed us to remove another potential confounder: the tail of the spermatozoon, which remains associated with one of the first two blastomeres after natural fertilization (Davies and Gardner, 2002; Jefferson and Williams, 2012). It is not clear if the tail may have an effect on the developmental processes (by engaging cytoplasmic processes of the host blastomere), but assuming that it would, then this difference between the sister blastomeres would not exist in mouse ICSI because the sperm tail is discarded prior to injection. We also examined the two sister blastomeres in the absence of mutual influence, by extracting them from the zona pellucida (splitting) and culturing them apart, thereby removing yet another confounder—the zona. The shape of the zona may influence the allocation of blastomeres’ progenies to the cell lineages of the blastocyst (Kurotaki *et al.*, 2007). When extracting the blastomeres from the zona, the second polar body remained trapped inside. This aspect may be relevant to a proposal that the blastomere to which second polar body is attached has greater potency than the other blastomere (Jin *et al.*, 2022). However, this proposal contrasts with a study conducted many years earlier, according to which the portion of the zygote that contained the second polar body could be cut off, and these zygotes still developed successfully to term (Zernicka-Goetz, 1998). As far as we are concerned, we note that our bisection procedure is such that the second polar body did not remain attached to a blastomere, but was trapped inside the zona.

Thanks to the precautions above, we characterized the nature of the epiblast imbalance more thoroughly than before. Immunofluorescence analysis of the three cell lineages at the blastocyst stage clearly showed that the epiblast cell number was more different in twin and cotwin when they originated from an oocyte fertilized at the equatorial site. The epiblast imbalance was not a transient trait, which perhaps would disappear on its own if we just examined the blastocyst stage at a later time point. As we identified the pluripotent/epiblast cells using the Oct4-GFP live-cell reporter and ‘immortalized’ them in the form of ES cells, we observed that the ES cell formation was higher from one twin than from the other when fertilization had occurred at the oocyte’s equator. In contrast to the protein and functional markers, mRNA markers were not as informative: the transcripts characteristically expressed in the epiblast showed no differences between twins and cotwins even though these differences existed (cell counts), whereas the transcripts of gene functions related to chromosome segregation were affected even though the total cell counts were not affected. Given these discrepancies between transcripts and phenotypes, we suggest that a transcriptional change is not necessarily consequential, owing to post-transcriptional gene regulation. Indeed, one of the data analyses we conducted revealed that GO terms related to ribosomes and protein translation were overrepresented among the genes with larger inter-blastocyst differences (consistent with a previous report, Zheng *et al.*, 2016). There are various lessons to be learned, including that if we had limited ourselves to transcript analysis, we would not have noticed the positional effect of fertilization on the epiblast imbalance at the blastocyst stage.

The crucial question is how the site of fertilization would lead to blastomeres of different properties, especially in the epiblast production. Research has been dealing with the origin of blastomere diversification for a hundred years, and we do not have the ambition to give an ultimate answer in this study, but we think our results can help to get closer to it. Previous work by others had favored stochastic or regulative origins of blastomere differences (Zernicka-Goetz, 2006; Dietrich and Hiiragi, 2007; Tang *et al.*, 2011; Bruce, 2013; Shi *et al.*, 2015). Because our experimental approach is positional (site-specific ICSI) and fertilization at the equator produces the most epiblast imbalance, there is no simple way to explain the results other than through differences in the molecular composition of the ooplasm, which, moreover, should also be apportioned variably at the first zygotic division. This possibility was already envisioned by Friedrich Seidel back in the 1950–1960s (Seidel, 1952, 1960), and relies on the variable orientation of the first cleavage axis. Although cleavage axes orient relative to a multiplicity of cues that have different strengths and competitive hierarchal relationships with one another, it should be possible to influence their behavior in such a way as to give one cue an edge over the others, as has been done, for example, when mouse zygotes were compressed laterally so as to force division along the shorter diameter (Gray *et al.*, 2004). In our case, the cue was the imposition of the fertilization site. We did not release the oocytes upon the ICSI at the A or V pole or the equator, but kept them immobilized for 24 h. This gave us the opportunity to record the orientation of the first zygotic cleavage relative to the A–V axis of the MII oocyte to see whether there was anything special when cleavage followed the equatorial ICSI. We observed that the oocytes fertilized by ICSI at the A (ipsilateral) or V pole (contralateral) had a bias to divide perpendicularly to the A–V axis, whereas the oocytes fertilized by ICSI at the equator were distributed in all possible ways. This equatorial variability is reminiscent of observations made by other scientists after polar material was removed from a mouse zygote by cuts made in different positions along the A–V axis: ‘cutting eggs perpendicularly to the A–V axis caused more of them (56%) to change the cleavage plane than cutting meridionally (29%)’, suggestive of factors distributed along the equatorial region with roles in the orientation of the cleavage axis (Zernicka-Goetz, 1998).

Even if the region of fertilization makes a difference and hints at the presence of oocyte territories that influence embryogenesis, our study’s mechanistic insight would have been limited without pinpointing a molecular culprit. It is not known how molecular gradients are distributed in the ooplasm, with a general assumption that they are distributed along the A–V axis, and that mitochondria are enriched at the A pole (Calarco, 1995; Li and Fan, 1997). However, Albert Dalcq and colleagues found in their study of rat oocytes that the boundary between the two distinct cytoplasmic territories they identified on the basis of histochemical reactivity was nearly parallel rather than orthogonal to the A–V axis (Dalcq, 1957, discussed in Denker (1983) and Gardner (1999)). A handful of proteins have been reported in more modern studies to be distributed non-homogeneously in the oocyte (LEPTIN and STAT3, Antczak and Van Blerkom, 1997; BCL-X, BAX, TGFΒ2, VEGF, and EGF-R, Antczak and Van Blerkom, 1999; PAR3 and PAR6, Duncan *et al.*, 2005 and Vinot *et al.*, 2004; and PAR4, Szczepanska and Maleszewski, 2005). Distributions of molecules in the ooplasm seem variable and irregular, not to say irreproducible, and the jury is still out when it comes to demonstrate clear-cut polarities. Caution is warranted when the detection method is whole-mount immunofluorescence, which can produce artefacts (Littwin and Denker, 2011). With the advent of genome-wide omics studies, a study from 2011 revealed asymmetries in the transcriptome composition within zygotes but not between sister blastomeres (VerMilyea *et al.*, 2011), while a more recent study found consistent asymmetries in the proteome both within zygotes and between sister blastomeres (Iwamoto-Stohl *et al.*, 2025). Assuming that non-homogeneity is there but transcript analysis is unsuited for detection, we decided to bisect the oocytes along both the A–V axis and the equator, and examine the protein composition of the two hemispheres using half-cell proteomics. The problem of distinguishing the two parts arose again, which was easy to solve when doing the equatorial bisection, but not when doing the meridional bisection. For this reason, we used an analysis method based on an analysis of variance, which did not rely on the exact categorization of the sister hemispheres, but only required us to know which hemispheres belonged together (the same oocyte origin). Furthermore, this method of analysis of variance is less sensitive to the volumetric error introduced by mechanical bisection. The variance analysis revealed that there are differences well over the 10% introduced by volumetric bisection between the A and V hemispheres as well as between the L and R hemispheres, and the former are preponderant. Thus, when the oocyte divides with variable orientation depending on the fertilization site, a variable partition of maternal proteins in the first blastomeres follows, and probably sets the stage for the imbalance of epiblast production by the two blastomeres. This is the first time that systematic differences in oocyte hemisphere composition have been reported after proteome analysis in a mammal, while this approach had already been successful in *Xenopus*, facilitated by the generous size of the oocyte and the more prominent distinction between the A and V poles (Sindelka *et al.*, 2018). Our study was not designed to investigate the role of specific proteins. We limit ourselves to note that proteins previously reported as being non-homogeneously (or even asymmetrically) distributed in mouse or human oocytes were not detected in our dataset, with the sole exception of STAT3 (SS wo/SS tot = 0.13 and 0.51 in the case of meridional and equatorial bisection, respectively). The proteins differentially distributed between the hemispheres are associated with mitochondria and endomembrane systems. This is in line with what we hypothesized in our previous study, namely, that next to the currently known mechanisms of blastomere diversification ‘at least one more mechanism is at play, potentially related to the endomembrane system’ (Casser *et al.*, 2018).

The observations reported here are contingent on the use of the ICSI and the fact that the oocytes used to study the epiblast imbalance were not the same as those used to measure ooplasm non-homogeneity and first cleavage orientation. Regarding the ICSI, its conditions are non-natural conditions, and ICSI embryos are not identical to natural counterparts in mice (Giritharan *et al.*, 2010), although the differences are probably not in fundamental biological properties, else there would not be millions of offspring who were born from ICSI in medically assisted human reproduction. A previous ICSI study conducted in mice could not detect any differences in the total counts of trophectoderm and inner cell mass cells between embryos produced by ICSI and those created by NF or *in vitro* insemination (Piotrowska-Nitsche and Chan, 2013). The authors concluded that the effect of the sperm entry point on the first cleavage axis was not altered by the method of fertilization (Piotrowska-Nitsche and Chan, 2013), and we concur, because the way the ICSI was done in that study was the conventional one, in which the operator tends to avoid the spindle region and, thus, introduces a bias toward equatorial fertilization, which is the one prevalent in *in vitro* insemination and NF. Regarding the use of different oocytes to measure various endpoints, it is clear that mass spectrometry is destructive, therefore, the same oocytes cannot be used for further downstream analyses. In the case of mass spectrometry, there is the additional fact that, for bisection, it was necessary to pre-treat the oocytes with an agent (Latrunculin B) that disrupts the actin cytoskeleton, so as to be able to cut the oocyte without lysis. It is unlikely that the ooplasmic distribution of proteins changed in the short time taken by the procedure (minutes), but we cannot rule it out. In theory, we could have used the oocytes from the measurements of the first cleavage orientation to study epiblast imbalance as well. The problem is that only one single oocyte was examined per day, (which would exacerbate batch effects) and the culture conditions applied during imaging are suboptimal (which probably does not influence a mechanical process such as the first cell division, but probably influences cell lineage formation if the embryos are cultured long enough). Thus, no embryo in this study has both a recorded cleavage angle and a measured epiblast contribution outcome.

In conclusion, our study highlights the contributions of ooplasm non-homogeneity and site of fertilization in the genesis of epiblast imbalance observed at the blastocyst stage in mice. Our results allow for the reconciliation of the different views regarding the biology of the first two blastomeres in the preeminent mammalian model: the traditional view that the sister blastomeres are equivalent, and the growing one that they are not equivalent. Both views are supported, depending on how the oocyte was fertilized and, consequently, how it cleaved and how the blastomeres received maternal proteins (or other molecules) in balanced or unbalanced amounts. This is not to say that these upstream features alone are sufficient or decisive, as we know that the unfolding of mammalian embryonic development is regulative; divergence in potency between the two blastomeres is likely governed by a complex interplay of processes rather than a single event or mechanism. Yet, it means that there is an initial element of oocyte architecture, which wanes during development, yet, its legacy can still be recognized at the blastocyst stage if we use suitable methods of analysis (proteins instead of mRNAs). Our proposal contrasts with that of a recent study, according to which it is the spermatozoon that breaks the homogeneity of the ooplasm and the symmetry of the first two blastomeres (Iwamoto-Stohl *et al.*, 2025).

Thinking about the future, we propose that the spatial effect of the fertilization site on the epiblast production could be worth investigating also in humans. Research on the impact of the ICSI site on human embryo development has yielded mixed results. Some studies found no significant difference in fertilization rates or clinical outcomes when comparing sperm deposition sites near or far from the meiotic spindle (Nagy *et al.*, 1995; Stoddart and Fleming, 2000). The tissue composition of human blastocysts (trophectoderm, primitive endoderm, and epiblast) has been investigated more often with mRNA markers (Durruthy-Durruthy *et al.*, 2016; Petropoulos *et al.*, 2016) than with protein markers (Niakan and Eggan, 2013), but, actually, we just learned from our results that transcript analysis is less apt to expose any differences. Nearly all human ICSI procedures are performed by injecting the spermatozoon at the equator (to avoid the meiotic spindle), effectively always using the ‘equatorial’ condition that this study found produced the largest epiblast imbalance in mice. Our mice were used for experiments at a maternal age of 8 weeks, which equates to human females in teenage years (Cottam *et al.*, 2025), whereas human oocytes fertilized by ICSI are typically from older women—in their thirties. The one study so far that documented the blastomere imbalance of human 2-cell embryos did not specify how old the women were (Junyent *et al.*, 2024). At any rate, for maternal age to have an influence on the imbalance, the distribution of proteins in the oocyte would need to change, or the orientation of the first zygotic division would need to change with maternal age, or both. The orientation of the first zygotic division was shown to matter in a study conducted on human IVF embryos that developed to term: embryo quality varied with said orientation (Porokh *et al.*, 2024). Granted, mouse and human oocyte architectures can differ, and a phenomenon observed in mouse embryos may not translate directly to humans. However, at least in the case of the epiblast imbalance, human-based observations (Junyent *et al.*, 2024) are consistent with those from mice (this study, and Casser *et al.*, 2017). Without questioning that mammalian embryonic development is predominantly regulative, it is probably also true that the architecture of the oocyte has a long-range influence on the blastocyst.

## Supplementary Material

gaag004_Supplementary_Data

## Data Availability

All data needed to evaluate this article are provided in the article or in the Supplementary Information. The filtering steps of the RNA-seq data analysis are summarized in Supplementary Table S1. The RNA-seq raw data are available via NCBI Gene Expression Omnibus with identifier GSE241089. For convenience, the complex information embedded in GSE241089 has also been simplified and summarized in the Supplementary Tables S3 and S4. The blastocysts’ cell counts are presented in Supplementary Tables S2, S5, and S6. The derivation of ES cells from twin blastocysts is presented in Supplementary Table S7. The angles of first zygotic cleavage are presented in Supplementary Table S8. The mass spectrometry proteomics data have been deposited in the ProteomeXchange Consortium via the PRIDE repository (http://proteomecentral.proteomexchange.org), with the dataset identifier PXD067319. For convenience, the complex information embedded in PXD067319 has also been simplified and summarized in Supplementary Table S9. Digital object identifiers of the tables are provided in the Supplementary Information.
