# Supplementary material for: Variable partition of non-homogeneous ooplasm sets the stage for divergent potency of 2-cell stage blastomeres
Source: Mol Hum Reprod. 2026 Feb 4;32(1):gaag004. doi: 10.1093/molehr/gaag004 (PMC13098151; doi:10.1093/molehr/gaag004)
Supplement: gaag004_Supplementary_Data [file gaag004_Supplementary_Data.zip › Nolte et al_supplementary materials_R2.pdf]

Supplementary information files

Variable partition of non-homogeneous ooplasm sets the stage for divergent potency of 2-cell stage blastomeres

Thomas Nolte, Reza Halabian, Steffen Israel, Yutaka Suzuki, Hannes C.A. Drexler, Wojciech Makalowski, Georg Fuellen and Michele Boiani

Supplementary Figure S1. Analysis of the transcriptomes of intact ICSI blastocysts.
Supplementary Figure S2. Transcript levels of *Actb* in intact vs. twin ICSI blastocysts as criterion used for the a-b ranking of twin blastocysts.
Supplementary Figure S3. Volcano plots of the transcriptomes of twin and cotwin blastocysts.
Supplementary Figure S4. Negative controls of CDX2, SOX17 and NANOG immunostaining.

Supplementary Table S1. Filtering steps of RNA-seq data.
Supplementary Table S2. Total cell counts of the blastocysts used as controls.
Supplementary Table S3. TPM of all genes detected twin and intact ICSI blastocysts.
Supplementary Table S4. TPM of cell lineage genes detected in twin ICSI blastocysts.
Supplementary Table S5. Cell lineage counts of intact and twin blastocysts after triple immunofluorescence (CDX2, SOX2, NANOG).
Supplementary Table S6. Δ cell lineage vs Δ total cells in ICSI and NF blastocysts.
Supplementary Table S7. Derivation of ES cells from twin blastocysts after ICSI and NF.
Supplementary Table S8. Angles of 1st zygotic cleavage after ICSI.
Supplementary Table S9. Proteomic analysis of bisected oocytes.

Supplementary figures

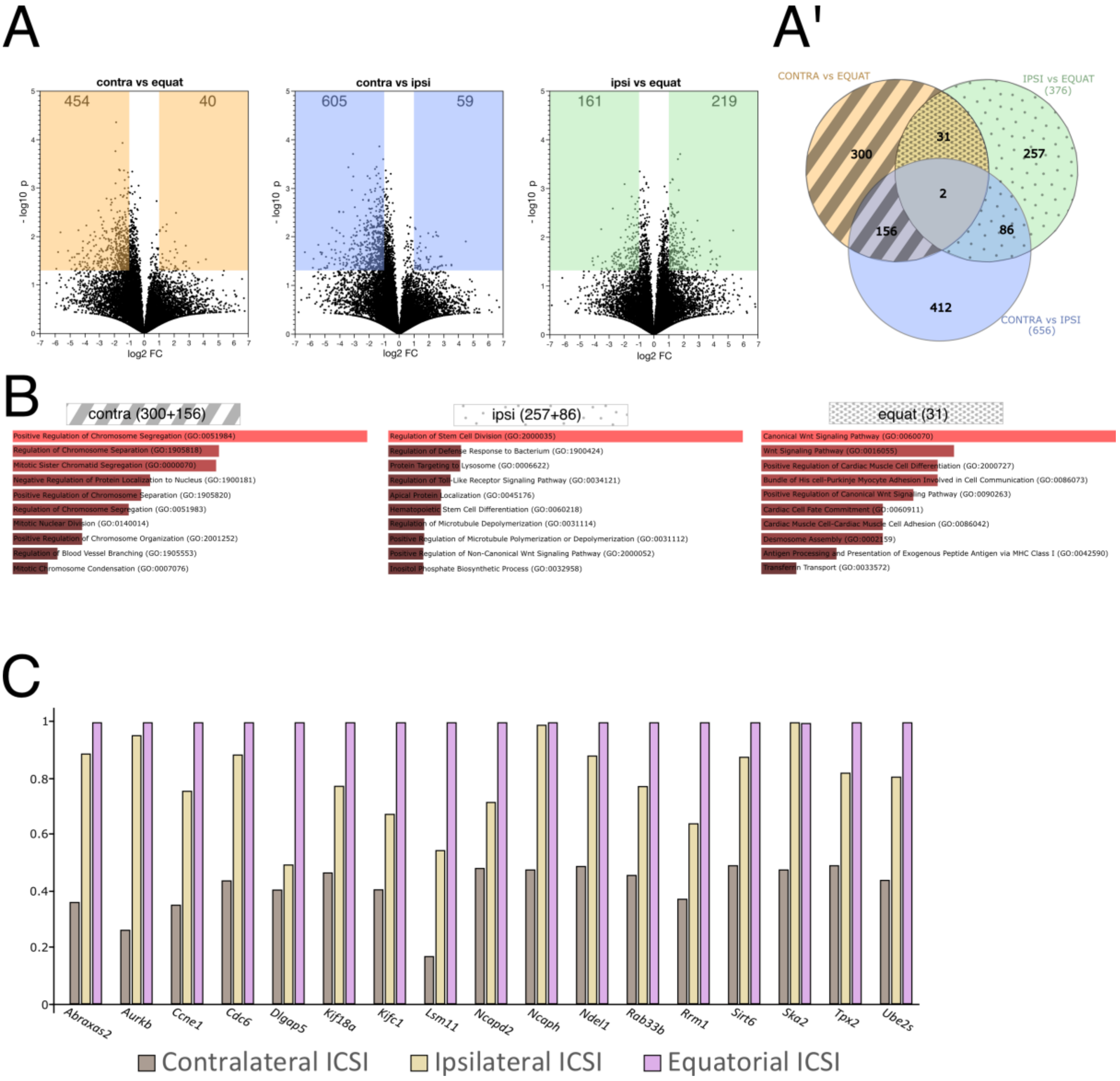

Supplementary Figure S1. Analysis of the transcriptomes of intact ICSI blastocysts. A. Shaded areas in Volcano plots include the data points of the differentially expressed genes, defined as ($\log_2 |FC| > 1$, - log 10 p value > 1.3). The numbers of differentially expressed genes are indicated in the corners of the shared areas. Within the differentially expressed gene sets, relevant subsets were identified by Venn diagram so as to be specific to the ipsilateral or contralateral or equatorial ICSI (hatched and dotted areas in **A'**). **B.** Bars show the results of overrepresentation analysis, performed with *Enrichr*, of the differentially expressed gene subsets that are specific to the ipsilateral or contralateral or equatorial ICSI. **C.** Normalized expression levels of the mRNA levels of genes involved in the stability of mitotic chromosomes (gene names retrieved from the relevant bar annotations in **B**). Volcano plots were generated using JMP. Venn diagram was generated using InteractiVenn (Heberle *et al.*, 2015). Bar plots were generated using *Enrichr* (Chen *et al.*, 2013; Kuleshov *et al.*, 2016; Xie *et al.*, 2021). Abbreviations: FC, fold change; p, probability value (Student's t test).

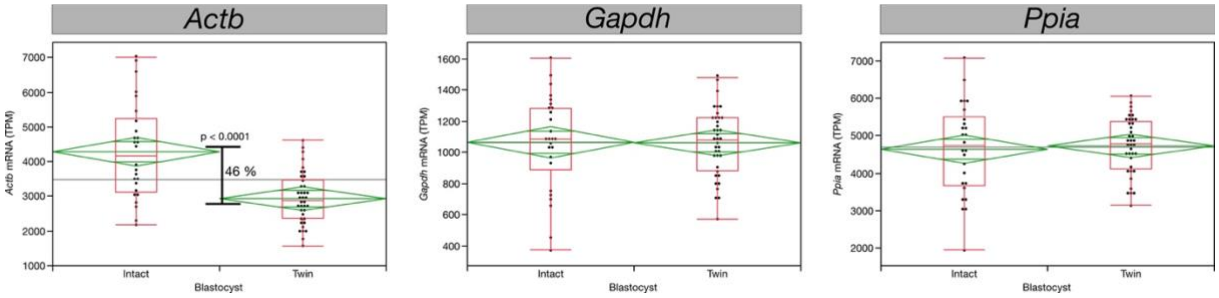

Supplementary Figure S2. Transcript levels of *Actb* in intact vs. twin ICSI blastocysts as criterion used for the a-b ranking of twin blastocysts. We show that the *Actb* mRNA level of intact embryos is on average 46 % significantly higher than that of 'half' (twin) embryos (Wilcoxon test), in line with the report that the mRNA of *Actb* correlates positively with the number of cells in the preimplantation mouse embryo (Giebelhaus *et al.*, 1983; Giebelhaus *et al.*, 1985). To be more confident that the relationship is specific to *Actb*, we also compared two other housekeeping genes, *Gapdh* and *Ppia,* that are expressed at approximately at about the same TPM level as *Actb.* The levels of *Gapdh* and *Ppia* mRNA were the same in the two types of embryos, thereby confirming that the relationship between embryo cell number and actin level is reliable. Therefore, we used this information to consistently assign the member of each blastocyst pair to the X-Y scatter plot axes in Figure 3, with the lower value assigned to the X axis (twin 'a') and the higher value assigned to the Y axis (cotwin 'b'). Abbreviations: *Actb, Actin b; Gadph, Glyceraldehyde-3-phosphate dehydrogenase; Ppia, Peptidylprolyl isomerase A*; TPM, transcripts per million.

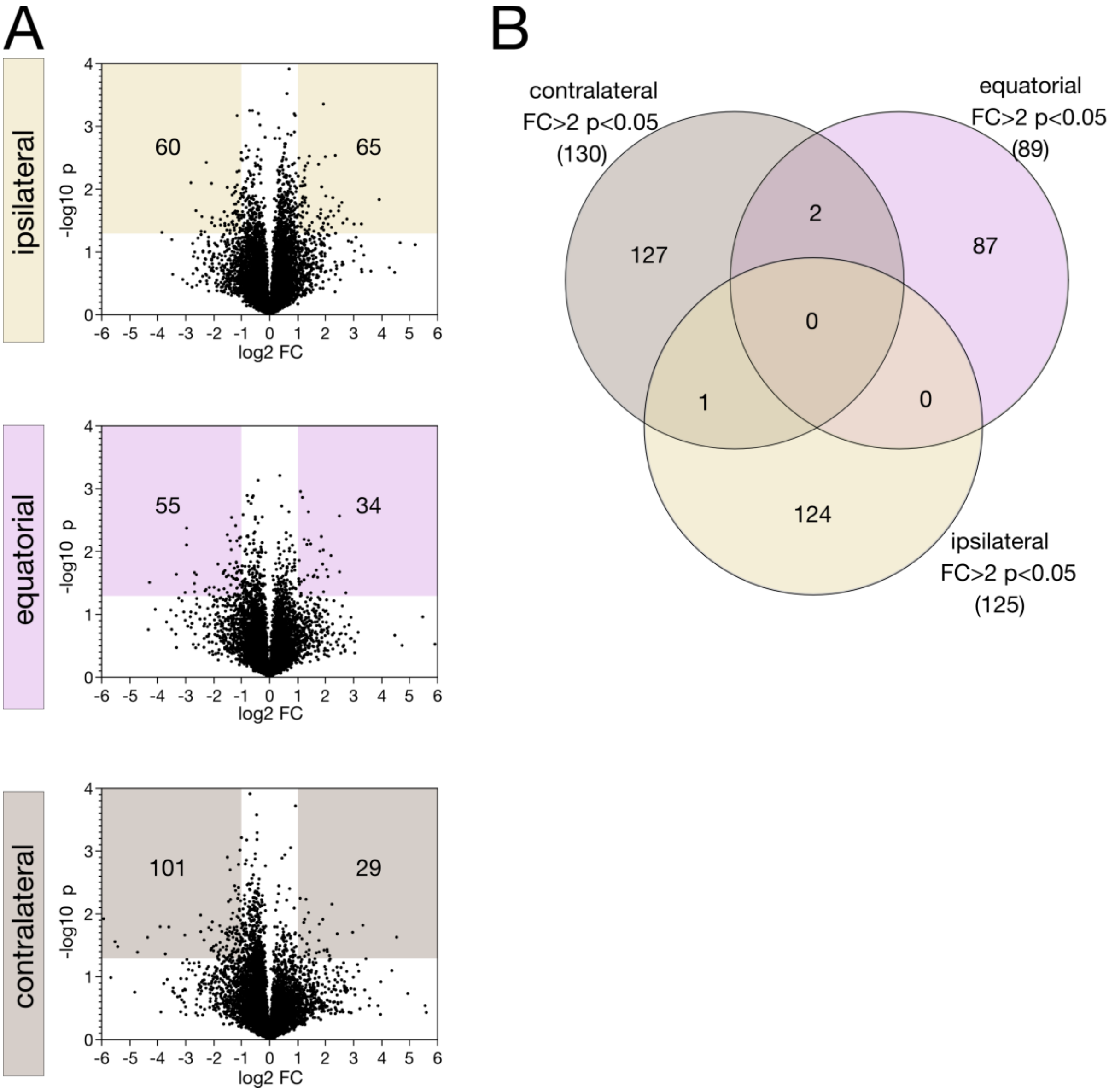

Supplementary Figure S3. Volcano plots of the transcriptomes of twin and cotwin ICSI blastocysts. A. Shaded areas in each Volcano plot include the data points of the differentially expressed genes, defined as (log_2 |FC| >1, -log 10 p value > 1.3). **B.** Differentially expressed genes are not shared between the three ICSI groups. Volcano plots were generated using JMP. Venn diagram was generated using InteractiVenn (Heberle *et al.*, 2015). Abbreviations: FC, fold change; p, probability value (Student's t test).

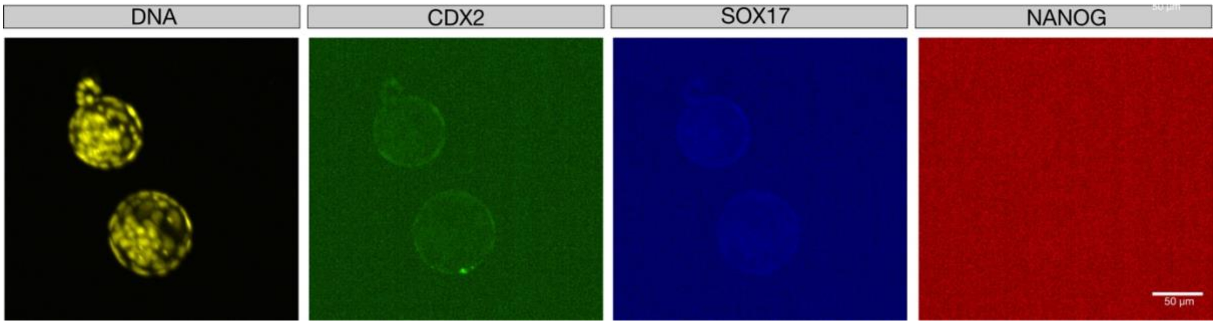

Supplementary Figure S4. Negative controls of CDX2, SOX17 and NANOG immunostaining. Specimens were processed as described in "Analysis of cell lineage allocation of blastocysts", except that the primary antibodies were omitted. No signal is observed in the areas corresponding to the cell nuclei's DNA (stained with Hoechst 33342, pseudocolored yellow).

Supplementary tables

Supplementary Table S1. Filtering steps of RNA-seq data.
https://doi.org/10.6084/m9.figshare.29376215

Supplementary Table S2. Total cell counts of the blastocysts used as controls.
https://doi.org/10.6084/m9.figshare.29268950

Supplementary Table S3. TPM of all genes detected twin and intact ICSI blastocysts.
https://doi.org/10.6084/m9.figshare.29268977

Supplementary Table S4. TPM of cell lineage genes detected in twin ICSI blastocysts.
https://doi.org/10.6084/m9.figshare.29268980

Supplementary Table S5. Cell lineage counts of intact and twin blastocysts after triple immunofluorescence (CDX2, SOX2, NANOG).
https://doi.org/10.6084/m9.figshare.29268962

Supplementary Table S6. Δ cell lineage vs Δ total cells in ICSI and NF blastocysts.
https://doi.org/10.6084/m9.figshare.29268968

Supplementary Table S7. Derivation of ES cells from twin blastocysts after ICSI and NF.
https://doi.org/10.6084/m9.figshare.29268983

Supplementary Table S8. Angles of 1st zygotic cleavage after ICSI.
https://doi.org/10.6084/m9.figshare.29268989

Supplementary Table S9. Proteomic analysis of bisected oocytes.
https://doi.org/10.6084/m9.figshare.29875349
