# Supplementary figures and images for: Variable partition of non-homogeneous ooplasm sets the stage for divergent potency of 2-cell stage blastomeres
Source: Mol Hum Reprod. 2026 Feb 4;32(1):gaag004. doi: 10.1093/molehr/gaag004 (PMC13098151; doi:10.1093/molehr/gaag004)

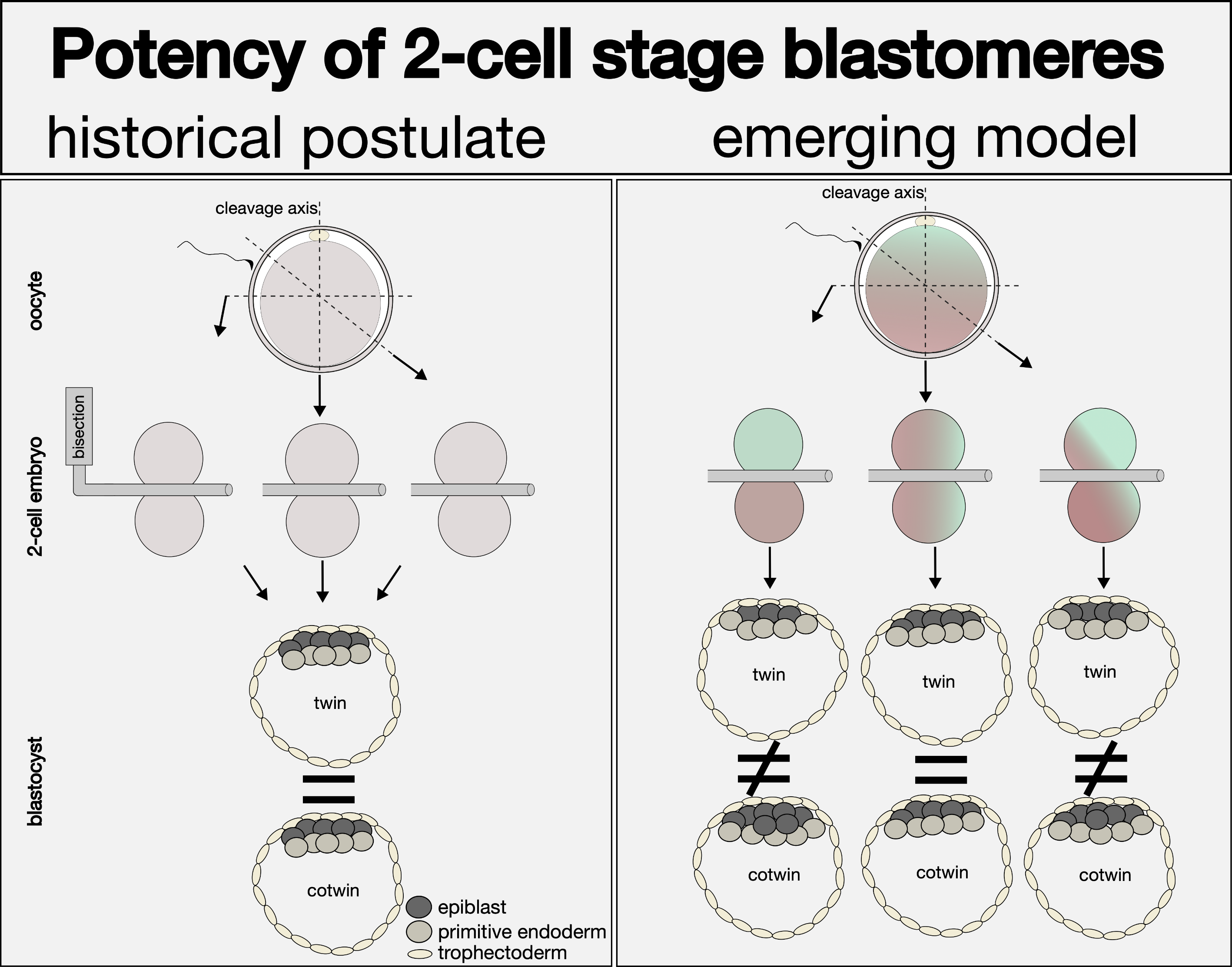

Supplement: gaag004_Supplementary_Data [file gaag004_Supplementary_Data.zip › graphical abstract_Nolte.tiff]
